# VPAC1 and VPAC2 Receptor Heterozygosity Confers Distinct Biological Properties to BV2 Microglial Cells

**DOI:** 10.3390/cells14110769

**Published:** 2025-05-23

**Authors:** Xin Ying Rachel Song, Margo Iris Jansen, Rubina Marzagalli, Giuseppe Musumeci, Velia D’Agata, Alessandro Castorina

**Affiliations:** 1 Laboratory of Cellular and Molecular Neuroscience, School of Life Sciences, Faculty of Science, University of Technology Sydney, Sydney, NSW 2007, Australia; raychelsxy@hotmail.com (X.Y.R.S.); margo.jansen@student.uts.edu.au (M.I.J.); rubina.marzagalli@uts.edu.au (R.M.); 2 Department of Biomedical and Biotechnological Sciences, Section of Anatomy, Histology and Movement Sciences, University of Catania, 95100 Catania, Italy; g.musumeci@unict.it (G.M.); vdagata@unict.it (V.D.)

**Keywords:** microglia, BV2 cells, VPAC1, VPAC2, PACAP, VIP, ER stress, unfolded protein response, neurodegeneration, neuroinflammation

## Abstract

Microglial cells, the resident immune cells of the central nervous system (CNS), are essential for maintaining CNS homeostasis. Dysregulation of microglial function is implicated in the pathogenesis of various neurodegenerative diseases. Vasoactive intestinal polypeptide receptors 1 and 2 (VPAC1 and VPAC2) are G-protein-coupled receptors (GPCRs) expressed by microglia, with their primary ligands being pituitary adenylate cyclase-activating polypeptide (PACAP) and vasoactive intestinal peptide (VIP). However, the specific roles of VPAC-type receptors in microglial regulation remain poorly understood. In this study, we generated VPAC1^+/−^ and VPAC2^+/−^ BV2 microglial cell lines using CRISPR-Cas9 gene editing and conducted a series of biological and molecular assays to elucidate the functions of these receptors. Our findings demonstrated that both mutant cell lines exhibited a polarized phenotype and increased migratory activity. VPAC1^+/−^ cells showed enhanced survivability and baseline activation of the unfolded protein response (UPR), a protective mechanism triggered by endoplasmic reticulum (ER) stress, whereas this response appeared impaired in VPAC2^+/−^ cells. In contrast, under lipopolysaccharide (LPS)-induced inflammatory conditions, UPR activation was impaired in VPAC1^+/−^ cells but restored in VPAC2^+/−^ cells, resulting in improved survival of VPAC2^+/−^ cells, whereas VPAC1^+/−^ cells exhibited reduced resilience. Overall, our findings suggest that VPAC1 and VPAC2 receptors play distinct yet complementary roles in BV2 microglia. VPAC2 is critical for regulating survival, ER stress responses, and polarization under basal conditions, while VPAC1 is essential for adaptive responses to inflammatory stimuli such as LPS. These insights advance our understanding of microglial receptor signaling and may inform therapeutic strategies targeting microglial dysfunction in neurodegenerative diseases.

## 1. Introduction

Neuroinflammation within the central nervous system (CNS) is a process predominantly regulated by two types of resident glial cells: microglia and astrocytes [1,2]. Although neuroinflammation is often perceived negatively and associated with CNS damage, its acute responses constitute a critical defensive mechanism designed to protect the brain from pathogens and/or injury while striving to restore homeostasis [1,2]. In contrast, chronic CNS inflammation leads to the sustained release of neurotoxic inflammatory mediators, reactive oxygen species, and reactive nitrate species, all of which contribute to a deleterious microenvironment that impairs cellular function and CNS healing processes [3,4]. Consequently, chronic neuroinflammation is widely recognized as a significant contributor to the pathogenesis of various neurodegenerative and traumatic neurological disorders, including multiple sclerosis (MS) [5], Alzheimer’s disease [6], Parkinson’s disease [7], amyotrophic lateral sclerosis (ALS) [8], and traumatic brain injury (TBI) [9].

Pituitary adenylate cyclase-activating polypeptide (PACAP) and vasoactive intestinal peptide (VIP) are neuropeptides extensively distributed within the CNS [10,11]. These neuropeptides share structural similarities and exhibit a wide range of biological activities, including neuroprotection and anti-inflammatory effects [12,13]. Their actions are mediated via three G-coupled receptors (GPCRs): the pituitary adenylate cyclase activating polypeptide receptor 1 (PAC1), vasoactive intestinal polypeptide receptor 1 (VPAC1), and vasoactive intestinal polypeptide receptor 2 (VPAC2), which mediate distinct cell-specific activities [14,15,16,17]. Notably, PACAP has more than 100-fold greater affinity for the PAC1 receptor compared to VIP, yet both peptides bind with high affinity to VPAC1 and VPAC2 receptors [10,15,18]. These differences suggest that PACAP/VIP receptor-specific biological effects largely depend on factors such as cell type, local receptor density, and localization [19,20].

VPAC1 and VPAC2 receptors are encoded by the *VIPR1* and *VIPR2* genes, respectively. These receptors are abundantly expressed in both the CNS and peripheral nervous system (PNS) [21]. Studies using VPAC1^−/−^ mice subjected to experimental autoimmune encephalomyelitis (EAE)—a murine model of MS [22]—demonstrated a delay in disease onset [23]. These findings suggested that *VIPR1* deficiency impairs CNS upregulation of chemokines and reduces the invasion of inflammatory cells from the periphery. In contrast, VPAC2^−/−^ mice subjected to EAE displayed exacerbated clinical and histopathological features, accompanied by impaired regulatory T cell (Treg) function [24]. These contrasting findings suggest that VPAC1 and VPAC2 receptors exert opposing functions during the effector phase of EAE, with VPAC1 primarily facilitating the onset of neuroinflammation, while VPAC2 acts as a molecular brake that restrains the inflammatory response. Similarly, in an experimental model of temporal lobe epilepsy, Serpa and colleagues reported differential regulation of VPAC1 and VPAC2 expression in the affected hippocampus [25], further supporting distinct roles for each receptor subtype.

Despite these advancements, the mechanisms underpinning the contrasting biological activities of VPAC receptors in microglia remain poorly understood. Given that PACAP and VIP are regulators of several key microglial functions—as evident from studies involving PACAP-deficient mice [26]—a deeper understanding of the biological roles of VPAC1 and VPAC2 receptors in unstimulated and polarized microglia remains a critical unanswered question.

Importantly, understanding how altered VPAC signaling affects microglial behavior at baseline and under inflammatory stress may offer novel mechanistic insights into early microglial dysfunction during neurodegeneration [27]. These in vitro findings could guide future experiments testing VPAC receptor modulation in animal models of MS or ALS [28], particularly by evaluating whether receptor-specific alterations impact disease onset, progression, or microglial activation profiles in vivo.

To address this gap, we used Clustered Regularly Interspaced Short Palindromic Repeats-Cas9 (CRISPR-Cas9) gene editing technology to generate murine BV2 microglial cell lines with heterozygous deletions of the VPAC1 or VPAC2 receptor genes (VPAC1^+/−^ or VPAC2^+/−^ microglia). These modified cell lines were subjected to a comprehensive battery of biological and molecular assays under resting conditions and after acute immune challenge with lipopolysaccharide (LPS). Our aim was to determine whether VPAC1 or VPAC2 haploinsufficiency would influence key microglial activities. A further goal was to elucidate any underlying intracellular adaptive mechanisms activated after partial gene ablation.

## 2. Materials and Methods

### 2.1. Cell Culture

BV2 microglial cells were kindly provided by Dr Eryn Werry from the University of Sydney, Sydney, Australia. Cells were grown (37 °C, 5% CO_2_) in Dulbecco’s Modified Eagle Medium (DMEM) mixture F-12 Ham (DMEM/F-12) supplemented with 10% heat-inactivated Fetal Bovine Serum (FBS) and 1% Penicillin (100 IU/mL)/streptomycin (100 μg/mL) (Merck Life Science, Bayswater, VIC, Australia). To sensitize cells to the effect of an immune challenge, cells were serum-starved for 24 h prior to lipopolysaccharide (LPS) treatment (1 μg/mL; Merck Life Science, Bayswater, VIC, Australia) for 0, 6, 12, and 24 h under reduced serum conditions (DMEM/F12 nutrient mixture, 1% Fetal Bovine Serum, 1% Penicillin/streptomycin; Merck Life Science, Bayswater, VIC, Australia), as in prior work [29].

### 2.2. Generation of VPAC1^+/−^ and VPAC2^+/−^ BV2 Microglial Cell Lines

The all-in-one PX458 plasmid vector (Addgene, Watertown, MA, USA) was used for the generation of CRISPR-Cas9 expression plasmids containing VPAC1 or VPAC2 guide (g)-RNA sequences. gRNA primer sequences that were ligated to the CRISPR-Cas9 plasmid vector were designed using Benchling^®^ (https://www.benchling.com/, (accessed on 20 March 2025); San Francisco, CA, USA) and are listed in Table 1.

A detailed description of the protocol for the generation of our CRISPR-Cas9 expression vector and cell transfection is described in detail elsewhere [30]. Cells were seeded and transfected with X-tremeGENE™ HP DNA Transfection Reagent (Merck Life Science, Bayswater, VIC, Australia) in Opti-MEM media (Thermo Fisher Scientific, Scoresby, VIC, Australia) supplemented with purified plasmid and P3000 reagent (Thermo Fisher Scientific, Scoresby, VIC, Australia). Reagent control cells underwent similar transfection; however, the plasmid was omitted. At 24 h post-transfection, green fluorescent protein (GFP)-positive cells were isolated and single-cell sorted using a BD FACSMelody™ cell sorter (BD Biosciences, Sydney, NSW, Australia) into a 96-well plate containing full growth media (FGM, DMEM/F12 nutrient mixture, 10% Fetal Bovine Serum, 1% Penicillin/streptomycin; Merck Life Science, Bayswater, VIC, Australia). Thereafter, single-cell clonal populations were expanded for 2–3 weeks and progressively sub-cultured into larger vessels (i.e., 24- and 6-well plates) until yielding sufficient cells for downstream biological and molecular applications.

To validate successful gene editing, genomic DNA was isolated from expanded clonal populations using the ISOLATE II Genomic DNA Kit (Millennium Science, Mulgrave, VIC, Australia) according to the manufacturer’s instructions. Purified DNA was then amplified by end-point polymerase chain reaction (PCR) using Phusion green Hot Start II High-Fidelity PCR Master Mix (Thermo Fisher Scientific, Scoresby, VIC, Australia) using PCR sequencing primers (shown in Table 2) and Sanger sequenced by the Australian Genomic Research Facility (AGRF, Westmead, NSW, Australia) using primer sets outlined in Table 3. To determine the successful targeting and efficiency of CRISPR-Cas9-induced gene deletion, sequencing results from VPAC1- and VPAC2-transfected clones were analyzed using the computational bioinformatic tool TIDE (Tracking of Indels by Decomposition, https://tide.nki.nl/, last accessed on 13 April 2023).

### 2.3. RNA Interference and Transfection of BV2 Microglial Cells

To induce transient attenuation of VPAC1 and VPAC2 receptor gene expression in BV2 microglial cells, small interfering RNAs (siRNAs) targeting Vipr1 (VPAC1; Santa Cruz Biotechnology, Dallas, TX, USA; cat. n. sc-40282) and Vipr2 (VPAC2; Santa Cruz Biotechnology, cat. n. sc-40284) were introduced using the Lipofectamine™ RNAiMAX transfection reagent (Thermo Fisher Scientific, Scoresby, VIC, Australia), following the manufacturer’s protocol with minor modifications. Briefly, BV2 cells were seeded in 6-well plates at a density of 2 × 10^5^ cells per well in antibiotic-free DMEM supplemented with 10% fetal bovine serum and allowed to adhere overnight.

The next day, siRNA-lipid complexes were prepared by incubating 30 nM of either VPAC1-specific, VPAC2-specific, or non-targeting control siRNA (Silencer Select, Thermo Fisher Scientific, North Ryde, NSW, Australia) with 7.5 µL of RNAiMAX reagent in Opti-MEM™ reduced serum medium (Thermo Fisher Scientific) for 10 min at room temperature. The complexes were then added dropwise to the cells, ensuring uniform distribution. Cells were maintained under standard culture conditions (37 °C, 5% CO_2_) for 48 h post-transfection, after which knockdown efficiency was validated by real-time quantitative PCR (please see Figure S2A,B). Transfected cells were subsequently used for downstream gene expression analyses, including assessments of pro- and anti-inflammatory genes (Figure S3A–E) and baseline expression of genes related to the unfolded protein response activation (Figure S3F,G).

### 2.4. RNA Extraction, cDNA Synthesis, and Real-Time Quantitative Polymerase Chain Reaction

Total RNA was isolated with 1 mL using TRI-reagent, to which we added 100 µL of 1-Bromo-3-Chloropropane (Merck Life Science, Bayswater, VIC, Australia) and centrifuged at 12,000× *g* for 15 min at 4 °C. The aqueous phase was then precipitated with 500 µL of 2-Isopropanol (Merck Life Science, Bayswater, VIC, Australia) as described previously [31]. The resulting pellet was washed with 75% ethanol twice and air-dried. Total RNA (1 µg) was reverse transcribed into cDNA using the Tetro cDNA synthesis kit, as reported in our previous work [30]. Real-time quantitative polymerase chain reaction (RT-qPCR) to amplify our genes of interest was performed in a final volume of 10 µL, consisting of 3 µL of input cDNA, 0.4 µL of milliQ H_2_O, 5 µL of iTaq Universal SYBR green master mix (Bio-Rad, South Granville, NSW, Australia), and 0.8 µL of forward and reverse primers (Table 4) (final concentration = 400 nM, Merck Life Science, Bayswater, VIC, Australia). Reaction mixtures were loaded onto Hard-shell^®^ 96-well PCR plates (Bio-Rad, South Granville, NSW, Australia) and run using the CFX96 Touch™ Real-Time PCR Detection System (Bio-Rad, South Granville, NSW, Australia). PCR parameters were set as follows: (1) hot-start denaturation at 95 °C for 2 min, (2) annealing at 60 °C for 10 s, (3) extension at 72 °C for 10 s, (4) plate read, and (5) repeat steps 2 to 4 for 45 cycles. Melting curve settings were (1) 65 °C for 35 s, (2) plate read, and (3) repeat steps 1–2 60 times. After the run was completed, Ct values were exported, and relative fold changes were calculated using the ∆Ct method [32], using ribosomal protein S18 as the reference gene. Baseline fold changes for untreated wild-type (WT) microglia were set to 1. Primer specificity was determined by running a melting curve analysis at the end of each PCR amplification.

### 2.5. Western Blot

Radioimmunoprecipitation assay buffer (RIPA) supplemented with protease inhibitor (cOmplete Tablets, Mini EDTA-free, EASYpack; Merck Life Science, Bayswater, VIC, Australia) was used to extract proteins. Lysates were then sonicated using an ultrasonic probe set at 50% amplitude (30 s bursts; Qsonica Q125 Sonicator, Thermo Fisher Scientific, Scoresby, VIC, Australia). Protein quantification was performed using the Bicinchoninic acid assay (BCA) kit (Thermo Fisher Scientific, Scoresby, VIC, Australia) according to the manufacturer’s instructions.

Protein samples (20 µg) were prepared for denaturation in 4× Laemmli buffer (Bio-Rad, South Granville, NSW, Australia) containing ß-mercaptoethanol (Merck Life Science, Bayswater, VIC, Australia). Samples were then denatured at 70 °C for 10 min and separated via sodium-dodecyl sulfate (SDS)-polyacrylamide gel electrophoresis (SDS-PAGE) using a 4–20% Mini-PROTEAN^®^ TGX Stain-Free™ protein gel (Bio-Rad, South Granville, NSW, Australia). Resolved proteins were then transferred onto polyvinylidene fluoride (PVDF) membranes (Trans-Blot^®^ Turbo™, Transfer Pack, Bio-Rad, South Granville, NSW, Australia) using Trans-Blot^®^ Turbo™ system (Bio-Rad, South Granville, NSW, Australia). Membranes were washed 3 × 5 min with tris-buffered saline containing 0.1% Tween^®^-20 (TBST, Merck Life Science, Bayswater, VIC, Australia). Thereafter, membranes were incubated in blocking buffer (5% skim milk in TBST) for 1 h at room temperature (RT) and probed with the desired primary antibodies overnight on slow oscillation at 4 °C (the full list of antibodies is shown in Table 5). Following incubation, the primary antibody was removed from membranes through several washes in TBST, followed by a secondary antibody incubation step for 1 h at room temperature (RT). Finally, the membrane was washed to remove excess secondary antibodies and incubated with Clarity™ Western ECL Blotting Substrate (Bio-Rad, South Granville, NSW, Australia). Images were then acquired using the ChemiDoc™ MP System (Bio-Rad, South Granville, NSW, Australia). The intensity of bands was quantified using the ImageJ image analysis software (version 1.53k).

### 2.6. Immunocytochemistry

Cells were grown on pre-coated coverslips (Poly-L-Lysine; Merck Life Science, Bayswater, VIC, Australia) placed in a 12-well plate and seeded at a density of 5 × 10^4^ cells/well. Cells were starved overnight prior to LPS treatment (1 μg/mL) in reduced serum conditions (1% fetal bovine serum [FBS]) for 12 h. Thereafter, cells were fixed using 4% paraformaldehyde (Merck Life Science, Bayswater, VIC, Australia) for 15 min at RT. Following a permeabilization step (0.3% Triton-X100 in phosphate buffered saline [PBS]; Merck Life Science, Bayswater, VIC, Australia), non-specific binding was blocked using a 5% bovine serum albumin (BSA) solution (Merck Life Science, Bayswater, VIC, Australia) for 1 h at RT. Desired primary antibodies (Table 6) were probed overnight in a humidified chamber. Following overnight incubation, coverslips were incubated in tetramethylrhodamine (TRITC)- and fluorescein isothiocyanate (FITC)-conjugated secondary antibodies (raised against different species) for 1 h in the dark. Coverslips were mounted with VECTASHIELD^®^ Antifade Mounting Medium with DAPI (Abacus DX, Cannon Hill, QLD, Australia) onto SuperFrost gelatinized microscope slides (Hurst Scientific, Forrestdale, WA, Australia). Finally, coverslips were imaged with the Leica STELLARIS 8 confocal microscope using a 63.5× oil-immersion objective, and images were analyzed using ImageJ (Version 2.9.0).

### 2.7. Nitric Oxide (Griess Assay)

To determine relative changes in the levels of nitric oxide (NO) released in the culture media, BV2 microglial cells were seeded in a 96-well plate at a density of 2 × 10^4^ cells/well and treated or not with LPS (1 μg/mL) for 6, 12, and 24 h. NO levels in the supernatant were determined by adding the Griess reagent (100 µL, Merck Life Science, Bayswater, VIC, Australia) to an equivalent volume of supernatant for 15 min at RT in the dark and under sterile conditions. Absorbance was measured at 540 nm using the Tecan™ infinite M1000 Pro ELISA plate reader (Tecan, Männedorf, Switzerland).

### 2.8. Annexin V/Propidium Iodide Staining (Flow Cytometry)

To investigate the degree of apoptotic cell death at baseline or after an immune challenge, both WT, VPAC1^+/−^, and VPAC2^+/−^ microglial cells were assayed using the Dead Cell Apoptosis Kit (V13242, Thermo Fisher Scientific, Scoresby, VIC, Australia) according to the manufacturer’s instructions. Briefly, untreated cells or cells challenged or not with LPS (1 μg/mL) for 24 h were harvested and resuspended in 100 µL of Annexin binding buffer. A total of 5 µL of Alexa Fluor™ 488 Annexin V and 1 μL of propidium iodide (PI) (100 μg/mL; Thermo Fisher Scientific, Scoresby, VIC, Australia) were then added to each 100 µL of cell suspension and incubated for 15 min in the dark. Samples were analyzed using a BD LSRFortessa™ X-20 cell analyzer (Becton Dickinson, Macquarie Park, NSW, Australia) with fluorescence emission set at 530 nm and excitation at 575 nm. Data collected were analyzed using the FloJo™ software version 10.9.

### 2.9. Real-Time Assessment of Cell Motility (Wound Healing Assay)

To assess the mobility of VPAC1^+/−^ and VPAC2^+/−^ BV2 microglial cells under resting conditions or after an LPS challenge, we conducted live monitoring of cells that had been subjected to the scratch wound assay. For this purpose, cells were seeded in a 96-well plate a day prior to the experiments at high density (6 × 10^4^ cells/well) to create a dense layer of cells. To guarantee uniform scratches, these were made using a 96-pin IncuCyte WoundMaker tool (Sartorius, Göttingen, Germany) and followed by two quick washes with 100 μL PBS to remove any detached cells/debris from the wounded area. Desired media (normal growth media or supplemented with 1 μg/mL LPS) was then added into the designated wells. Cells were scanned every 3 h for a total of 96 h using the IncuCyte^®^ Live-Cell Analysis System (Sartorius, Göttingen, Germany). Analyses were performed using the Incucyte^®^ Scratch Wound Analysis Software Module version 2022B (Sartorius, Göttingen, Germany).

### 2.10. Statistics

Results yielded from molecular and biological assays were analyzed and plotted using GraphPad Prism^®^ 9.3. Statistical significances between two groups were analyzed using an unpaired Student’s *T*-test. For multiple comparisons (three or more groups)—after confirming normal data distribution using Q-Q plots—one-way ANOVA was used for the analyses of data from untreated cells of either genotype, whereas two-way ANOVA was employed to factor in both genotype and treatment and their interaction. Tukey post-hoc tests were used to determine statistical significance. Probability values of less than 0.05 were considered statistically significant. 

## 3. Results

### 3.1. Validation of VPAC1 and VPAC2 Receptor Heterozygosity in BV2 Microglia

**Figure 1:**
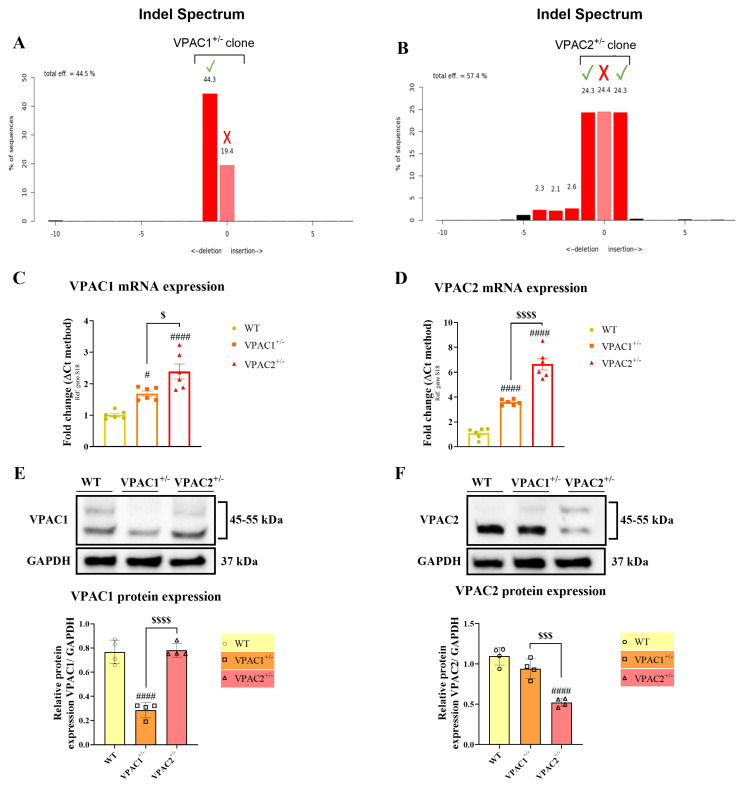
Validation of BV2 microglia VPAC1 and VPAC2 haploinsufficiency. TIDE indel spectrum analysis of the (**A**) VPAC1 and (**B**) VPAC2 clonal populations demonstrates that two out of three alleles were edited within each sequence. Edited alleles appear in red, whereas unedited ones are shown in pink. Bar graphs illustrate the mean fold change ± SEM (n = 6) of the mRNA expression of the (**C**) *VPAC1* and (**D**) *VPAC2* genes in the VPAC1^+/−^ and VPAC2^+/−^ BV2 microglial cells, as determined by RT-qPCR. Representative VPAC1 and VPAC2 blots and bar graphs displaying densitometric analyses of protein expression in both heterozygous cell lines (**E**,**F**). The results shown are the mean ± SEM of two experiments, each calculated by averaging two technical duplicates from four biological replicates (n = 4). # *p* < 0.05, or #### *p* < 0.0001 vs. WT. $ *p* < 0.05, $$$ *p* < 0.001, or $$$$ *p* < 0.0001 vs. VPAC1^+/−^, as computed using one-way ANOVA followed by the Tukey post-hoc test.

Using CRISPR/Cas9, we generated stable BV2 microglial cell clones harboring heterozygous gene deletions of the *Vipr1* and *Vipr2* genes (VPAC1^+/−^ and VPAC2^+/−^ cells). Evidence of effective transfection of BV2 microglia with the CRISPR-Cas9 expression vectors harboring either the VPAC1 or VPAC2 genes was made possible by taking advantage of the GFP construct present in the all-in-one PX458 plasmid (please refer to Figure S1A,B). Following transfection and clonal expansion, DNA samples obtained from mutant cells were Sanger sequenced to determine the successful placement of gRNAs within the desired genomic sequences and the consequent introduction of downstream frameshift mutations. Importantly, BV2 microglial cells have been immortalized via retroviral transduction with *v-raf*/*v-myc* oncogenes, and genome-wide inspections and sequencing analyses are strongly suggestive of aneuploidy, so it is not uncommon to identify chromosomes with potential trisomy or tetrasomy [30,33,34]. Analyses of the insertion/deletion (indel) spectrum using TIDE demonstrated the presence of two overlapping deletions of −2 base pairs (BPs) (in red) and 1 unaffected/wild-type (WT) allele (in pink) in the VPAC1^+/−^ clone, indicative of VPAC1 heterozygosity (Figure 1A). Analyses of VPAC2^+/−^ clones showed +1 BP insertion (red), −1 BP deletion (red), and a WT allele (pink), confirming VPAC2 heterozygosity (Figure 1B).

To further determine if the targeted mutations in our mutant cell lines affected gene transcription and significantly diminished protein translation, we conducted both real-time qPCRs (Figure 1C,D) and Western blots in each cell line (Figure 1E,F). We found that in VPAC1^+/−^ cells there was a significant increase in both *VPAC1* (# *p* < 0.05 vs. WT; Figure 1C) and *VPAC2* mRNAs (### *p* < 0.001; Figure 1C), with significant differences when comparing *VPAC1* and *VPAC2* mRNAs ($ *p* < 0.05 vs. VPAC1^+/−^ cells). A similar transcriptional dysregulation was observed in VPAC2^+/−^ cell lines, where both *VPAC1* and *VPAC2* transcripts were also upregulated (#### *p* < 0.0001 vs. WT; Figure 1D), with substantial differences between the two genes ($$$$ *p* < 0.0001 vs. VPAC1^+/−^ cells). This phenomenon is not uncommon when employing CRISPR-Cas9-based gene editing [35] and was not corroborated by protein expression data. In fact, Western blots revealed that VPAC1 protein expression was significantly decreased in VPAC1^+/−^ cells (### *p* < 0.001; Figure 1E), without affecting VPAC2 expression ($$$$ *p* < 0.0001 vs. VPAC1^+/−^ cells). Likewise, VPAC2 protein expression was significantly reduced in VPAC2^+/−^ cells (#### *p* < 0.0001; Figure 1F), but not in VPAC1^+/−^ cells ($$$ *p* < 0.0001 vs. VPAC1^+/−^ cells).

### 3.2. VPAC1^+/−^ and VPAC2^+/−^ BV2 Microglial Cell Lines Exhibit Increased Inflammatory Profiles

BV2 microglial cells express functional VPAC1 and VPAC2 receptors [29], both of which are implicated in regulating cytokine expression and release [36]. Here, we set out to explore the effects of VPAC1 or VPAC2 haploinsufficiency on the baseline gene expression levels of the pro-inflammatory cytokines (*interleukin-1β* [*IL-1β*] and *-6* [*IL-6*]), the microglial marker *ionized calcium-binding adapter molecule 1* (*Iba1*) [37], and the anti-inflammatory markers (*Arginase-1* and *CD206*) by real-time qPCR.

**Figure 2:**
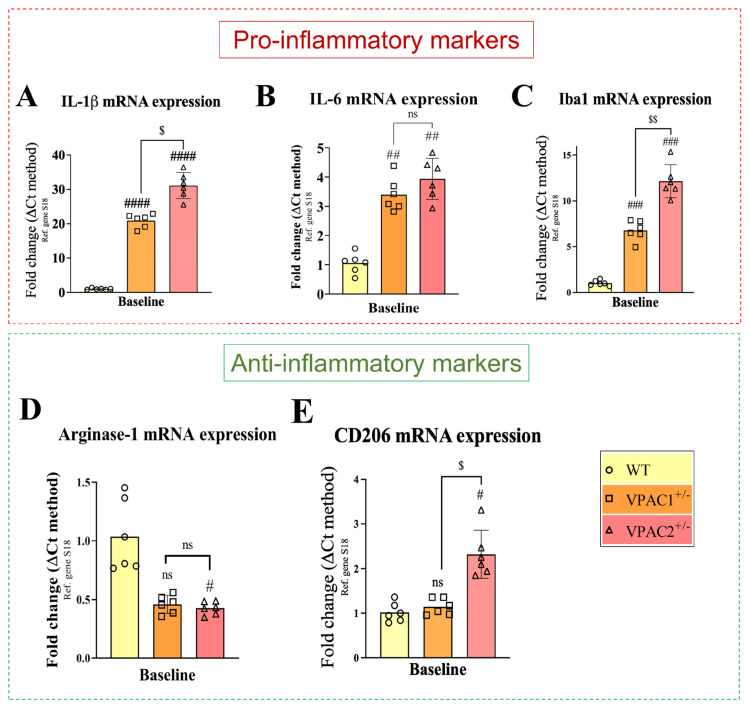
VPAC1 and VPAC2 haploinsufficiency induces a pro-inflammatory phenotype in BV2 microglia. Bar graphs depicting the mean fold change ± SEM in gene expression of the inflammatory markers (**A**) *IL-1β*, (**B**) *IL-6*, and (**C**) *Iba1* and the anti-inflammatory markers (**D**) *Arginase-1* and (**E**) *CD206* in WT, VPAC1^+/−^, and VPAC2^+/−^ cells. Data were obtained from six biological replicates, each performed using technical duplicates (n = 6). # *p* < 0.05, ## *p*< 0.01, ### *p* < 0.001, or #### *p* < 0.0001 vs. WT; $ *p* < 0.05 or $$ *p* < 0.01 vs. VPAC1^+/−^ cells, as determined by one-way ANOVA, followed by Tukey post-hoc analyses. ns = not significant.

*IL-1β* is a potent inflammatory cytokine responsible for mediating inflammatory cascades [38]. Likewise, *IL-6* is a pro-inflammatory cytokine produced by glial cells in response to inflammation [39]. As shown, partial loss of VPAC1 or VPAC2 both caused a robust increase in *IL-1β* (#### *p* < 0.0001 vs. WT; Figure 2A), *IL-6* (## *p* < 0.01; Figure 2B), and *Iba1* mRNAs (### *p* < 0.001; Figure 2C). In VPAC2^+/−^ cells, the increase in *IL-1β* transcripts was significantly higher than in VPAC1^+/−^ cells ($ *p* < 0.05; Figure 2A), and so was *Iba1* ($$ *p* < 0.01; Figure 2C), but not *IL-6* (*p* > 0.05).

Expression levels of anti-inflammatory genes were also analyzed in response to partial VPAC1 or VPAC2 gene deletion (Figure 2D,E). Expression of *Arginase-1*, an M2 microglial marker [40], showed a marginal reduction in both genotypes and was statistically significant only in VPAC2^+/−^ microglia (# *p* < 0.05 vs. WT; Figure 2D). In contrast, the expression of *CD206*, another anti-inflammatory marker [41], was significantly increased in VPAC2^+/−^ microglia both with respect to WTs (# *p* < 0.05) and VPAC1^+/−^ cells ($ *p* < 0.05; Figure 2E).

To confirm if the baseline effects seen in heterozygous cell lines could also be replicated in a cell system with transient *VPAC1* or *VPAC2* gene attenuation, additional experiments using silencing RNA (siRNA) were run in parallel. As shown in Figure S2, in comparison with untargeted siRNA controls, *VPAC1* and *VPAC2* siRNA-transfected BV2 cells exhibited robust reduction in both transcript (** *p* < 0.01 vs. Control siRNA for both, respectively; Figure S2A,B) and protein levels (*** *p* < 0.001 vs. Control siRNA for VPAC1 and ** *p* < 0.01 vs. Control siRNA for VPAC2, respectively; Figure S2C,D). Once confirmed, these cells were interrogated for the same genes as in haploinsufficient cells. Notably, when compared with non-targeting siRNA-transfected controls, changes in the expression of both pro- and anti-inflammatory genes mirrored those seen in VPAC1^+/−^ and VPAC2^+/−^ cells (Figure S3A–E).

**Figure 3:**
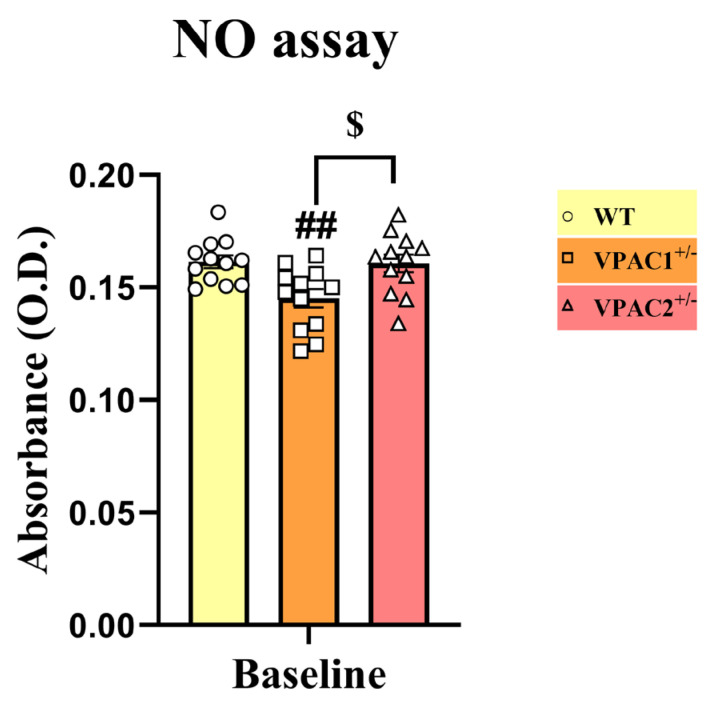
Partial VPAC1 but not VPAC2 gene loss in BV2 microglia reduces nitric oxide (NO) release. The bar graph demonstrates a significant reduction in NO release in the culture media of VPAC1^+/−^ but not in VPAC2^+/−^ microglia. The results shown are the mean ± SEM of three independent experiments, each using four separate biological replicates per genotype (n = 12). ## *p* < 0.01 vs. WT and $ *p* < 0.05 vs. VPAC1^+/−^ cells, as determined by one-way ANOVA, followed by Tukey post-hoc analysis. O.D. = optical density.

To corroborate gene expression studies, providing evidence of heightened levels of inflammatory markers in VPAC1^+/−^ and VPAC2^+/−^ cells, our next step was to employ the Griess reagent assay to determine if the relative levels of nitrates (NO) were also affected in mutant cells (and WT controls) (Figure 3). As shown, partial VPAC1 deletion caused a robust reduction in NO (## *p* < 0.01 vs. WT); however, VPAC2^+/−^ cells showed similar NO levels as WT (*p* > 0.05), which were significantly higher than in VPAC1^+/−^ cells ($ *p* < 0.05 vs. VPAC1^+/−^ cells, Figure 3).

### 3.3. VPAC1 and VPAC2 Haploinsufficiency Differentially Affects the Survivability of BV2 Microglial Cells

In this set of experiments, we investigated whether partial ablation of either VPAC1 or VPAC2 receptor genes differentially affected the basal survivability of BV2 microglial cell lines. To do so, we utilized a flow cytometry-based Annexin V/propidium iodide (PI) apoptotic assay.

**Figure 4:**
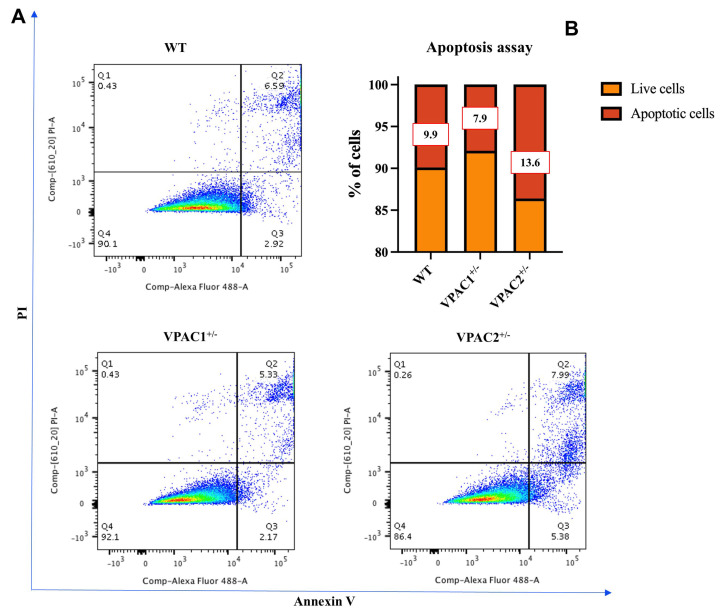
VPAC2^+/−^ haploinsufficiency reduces microglial cell viability. Both WT, VPAC1^+/−^ and VPAC2^+/−^ BV2 microglial cells were assessed for baseline apoptosis. (**A**) Apoptosis of BV2 cells was assessed by flow cytometric analysis using the Annexin V/propidium iodide (PI) staining. Live cells are found in the lower left quadrant (Annexin V and PI negative, Q4), early apoptotic cells are indicated in the lower right quadrant (Annexin V positive and PI negative, Q3), late apoptotic cells are demonstrated in the upper right quadrant (Annexin V and PI positive, Q2), and dead cells are shown in the upper left quadrant (Annexin V negative and PI positive, Q1). Each color represents the collection of events/cells with the same intensity detected during flow cytometry. Blue represents low intensity (single events/cells), whereas red represents high intensity. (**B**) Quantification of the total % of live/dead cells. Exact percentages of apoptotic cells are indicated for each genotype. Data shown are representative of three independent experiments.

Reagent control WT cells (i.e., unedited cells that had undergone the same CRISPR-Cas9 transfection process as mutant cells but without the addition of the plasmid to the transfection media) demonstrated some degree of baseline apoptosis (9.9%), probably due to the chemical and mechanical stress caused by the mock transfection (Figure 4A,B). Interestingly, partial VPAC1 gene deletion slightly improved baseline viability, as the total percentage of apoptotic cells decreased to 7.9% (−2% vs. WT cells). In contrast, VPAC2^+/−^ cells exhibited a higher baseline apoptotic rate (13.6%), with a +3.7% and +5.7% increase in the percentage of apoptotic cells in comparison with WT or VPAC1^+/−^ cells, respectively (Figure 4A,B).

### 3.4. VPAC1^+/−^ and VPAC2^+/−^ BV2 Microglia Show Opposite Baseline Activation of the Unfolded Protein Response

The partial loss of VPAC1 and VPAC2 receptors was found to increase and decrease the baseline survivability of BV2 microglia, respectively. In parallel, recent studies have highlighted a link between the PACAP/VIP neuropeptide system and the activation of various branches of the unfolded protein response (UPR) [42,43,44], a conserved mechanism that mitigates endoplasmic reticulum (ER) stress [45]. Based on these findings, we investigated whether partial ablation of *VPAC1* or *VPAC2* affects UPR activation in BV2 microglia.

**Figure 5:**
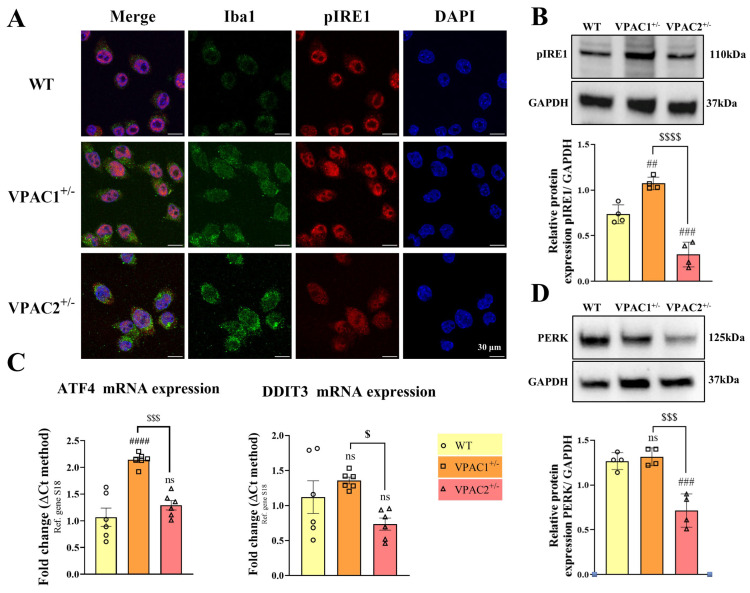
VPAC1 and VPAC2 heterozygosity oppositely regulate the unfolded protein response (UPR) in unstimulated BV2 microglia. (**A**) Representative confocal images showing Iba1-phospho-IRE1α^(Ser724)^ co-staining (pIRE1) in reagent control WT, VPAC1^+/−^, and VPAC2^+/−^ BV2 microglia. (**B**) Western blot of pIRE1 using lysates from the three different genotypes. Each experiment was reproduced four times (n = 4), and data were averaged after normalization to GAPDH. Results shown in bar graphs are the mean ± SEM. (**C**) Bar graphs depicting relative gene expression changes across genotypes ± SEM, as determined by real-time qPCR using n = 6 biological replicates per group. (**D**) Representative Western blots and semi-quantification of PERK protein expression ± SEM from four independent determinations (n = 4), normalized to GAPDH. ## *p* < 0.01, ### *p* < 0.001, or #### *p* < 0.0001 vs. WT and $ *p* < 0.05, $$$ *p* < 0.001, or $$$$ *p* < 0.0001 vs. VPAC1^+/−^ cells, as determined by one-way ANOVA and Tukey post-hoc test. ns = not significant, *ATF4* = activating transcription factor 4, *DDIT3* = DNA damage-inducible transcript 3, PERK = protein kinase R (PKR)-like endoplasmic reticulum kinase, and GAPDH = glyceraldehyde-3-phosphate dehydrogenase.

Qualitative assessments of the microglial marker Iba1 using immunohistochemistry suggested an increased cellular pro-inflammatory-like state in both mutant cell lines with respect to WT (Figure 5A), corroborating gene expression data shown previously in Figure 2.

Both immunohistochemistry and Western blot analysis demonstrated that VPAC1^+/−^ cells exhibited heightened phosphorylation of IRE1α at the serine 724 residue (## *p* < 0.01 vs. WT; Figure 5A,B). IRE1α is a transmembrane ER stress sensor, and phosphorylation of the serine 724 residue is essential for the activation of the UPR under ER stress [46]. In contrast, pIRE1 levels were significantly reduced in VPAC2^+/−^ cells, both in comparison to WT (### *p* < 0.001) and VPAC1^+/−^ microglia ($$$$ *p* < 0.0001).

Gene expression studies partly corroborated these findings, as baseline *ATF4* but not *DDIT3* gene expression levels were increased in VPAC1^+/−^ cells (#### *p* < 0.0001 vs. WT; Figure 5C) and unchanged in VPAC2^+/−^ cells (*p* > 0.05 vs. WT). However, the expression of both *ATF4* and *DDIT3* was reduced when comparing the two genotypes ($$$ *p* < 0.001 and $ *p* < 0.05 vs. VPAC1^+/−^ cells, respectively; Figure 5C). A similar pattern was observed when examining *DDIT3* and *ATF4* gene expression in BV2 microglial cultures treated with siRNA targeting *VPAC1* or *VPAC2*. Specifically, *ATF4* expression was significantly increased following *VPAC1* knockdown, whereas it remained unchanged in *VPAC2*-deficient cells (Figure S3F). In contrast, *DDIT3* expression remained unaffected by *VPAC1* knockdown but was markedly downregulated in *VPAC2*-silenced cells (Figure S3G).

Finally, analyses of PERK—another key element and upstream activator of the UPR [47]—showed that VPAC2^+/−^ microglia (but not VPAC1^+/−^) had reduced PERK protein expression both with respect to WT (### *p* < 0.001) and VPAC1^+/−^ cells ($$$ *p* < 0.001; Figure 5D).

### 3.5. Increased Baseline Mobility of VPAC1^+/−^ and VPAC2^+/−^ BV2 Microglia

Microglia are migratory cells whose mobility is crucial for the surveillance of the surrounding cellular microenvironment [48]. Here, using a live monitoring system, we assessed the mobility of WT and mutant cells after a scratch wound healing assay.

**Figure 6:**
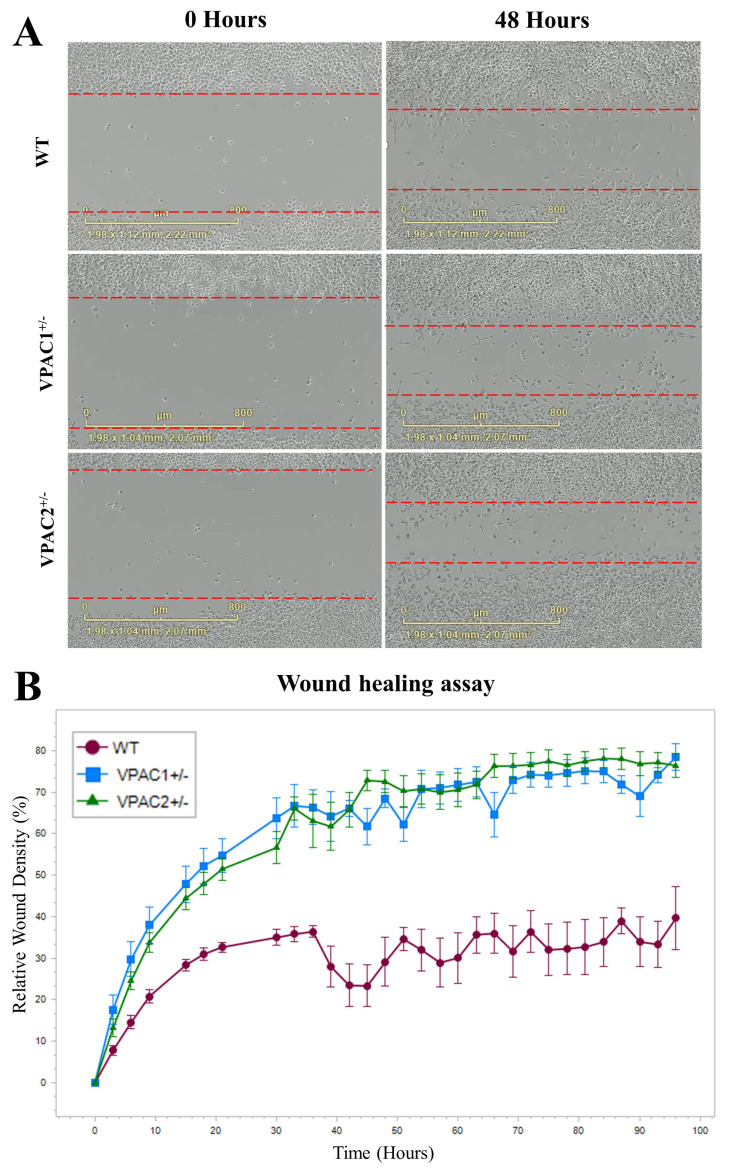
VPAC1 and VPAC2 haploinsufficiency increases BV2 microglial motility. (**A**) Representative images captured using the Incucyte Cell Imager comparing initial and final wound sizes at time 0 and after 48 h for each genotype. (**B**) Line graph obtained using the Incucyte Live Analysis software (version 2022B) depicting the time course of relative wound density (RWD) ± SEM in WT, VPAC1^+/−^, and VPAC2^+/−^ microglia. Wounds were scanned at regular intervals of 3 h over a period of 96 h. RWD measures the rate of wound closure compared to the initial scratch. This experiment was performed on six independent batches of cells per genotype (n = 6).

As shown in the representative images (Figure 6A), VPAC1^+/−^ and VPAC2^+/−^ cells had increased baseline motility compared to WTs. Live measurements of relative wound density (RWD)—defined as the cell density within the wound area relative to that outside the wound and used here as an indicator of cell motility—revealed a near-exponential increase in motility in the mutant cell lines during the first 30–36 h, followed by a plateau up to the final time point at 96 h. A similar but less pronounced pattern was observed in wild-type (WT) cells (Figure 6B). Data also showed a much faster wound recovery in both VPAC1^+/−^ and VPAC2^+/−^ cells than in WTs (RWD [VPAC1^+/−^ and VPAC2^+/−^ cells] = 60–70% vs. RWD [WT] = 30–35%, respectively (Figure 6B).

### 3.6. Partial Deletion of VPAC1 and VPAC2 Enhances Pro-Inflammatory Gene Expression in BV2 Microglia

**Figure 7:**
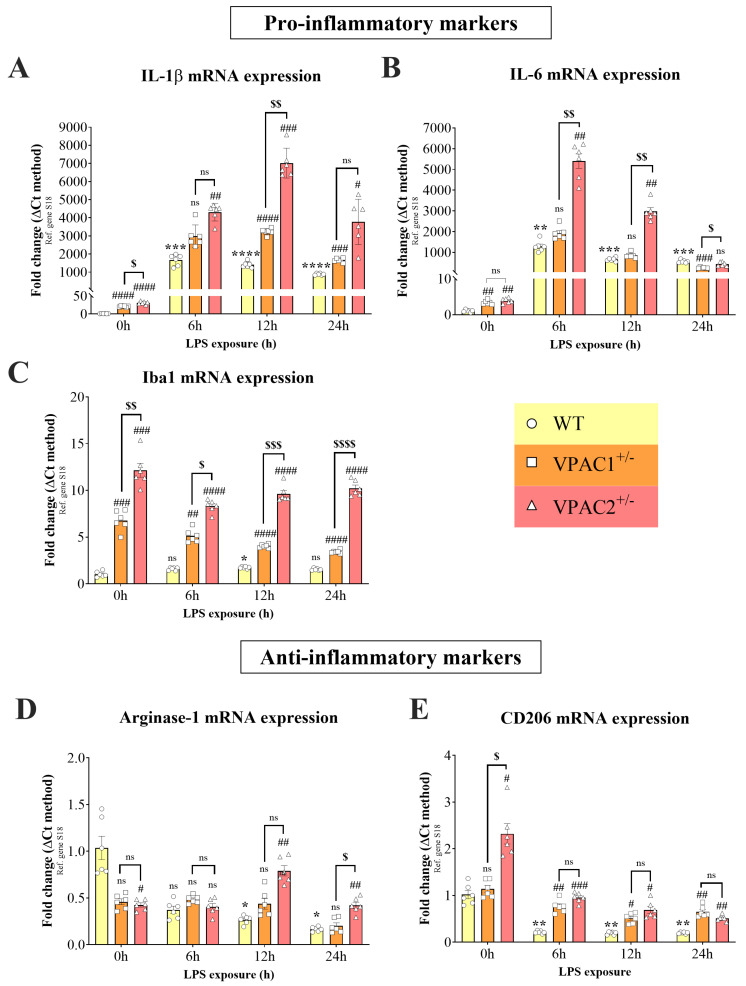
VPAC1^+/−^ and VPAC2^+/−^ microglia exhibit abnormally heightened expression of pro-inflammatory genes after an immune challenge with lipopolysaccharide (LPS). WT, VPAC1^+/−^, and VPAC2^+/−^ BV2 cells were stimulated with LPS for 0, 6, 12, and 24 h. Relative gene expression changes were measured by real-time qPCR, and relative fold changes were calculated using the ∆Ct method after normalization to the ribosomal protein subunit S18, which was used as the reference gene. Bar graphs illustrate the changes in the expression of the pro-inflammatory genes (**A**) *IL-1β*, (**B**) *IL-6*, and (**C**) *Iba1* and the anti-inflammatory genes (**D**) *Arginase-1* and (**E**) *CD206*. Results are presented as mean fold changes ± SEM values of three independent determinations, each using two biological replicates (n = 6). Baseline values of untreated groups were set to 1. * *p* < 0.05, ** *p* < 0.01, *** *p* < 0.001, or **** *p* < 0.0001 vs. WT at time 0. # *p* < 0.05, ## *p* < 0.01, ### *p* < 0.001, or #### *p* < 0.0001 vs. WT at the corresponding time point. $ *p* < 0.05, $$ *p* < 0.01, $$$ *p* < 0.001, or $$$$ *p* < 0.0001 vs. VPAC1^+/−^ cells, as determined using repeated measures two-way ANOVA, followed by Tukey post-hoc test. ns = not significant.

Building on the finding that partial loss of either VPAC1 or VPAC2 genes resulted in a gene expression profile indicative of a polarized state in BV2 microglia, the subsequent step aimed to evaluate the response of these mutant cell lines to an exogenous inflammatory stimulus, specifically lipopolysaccharide (LPS). Using real-time qPCR, we interrogated the same pro- and anti-inflammatory genes as in Section 3.2, but at different experimental time points (0, 6, 12, and 24 h, respectively) after administration of 1 µg/mL LPS, both in WT, VPAC1^+/−^, and VPAC2^+/−^ cells (Figure 7). We utilized this specific concentration of LPS based on prior evidence demonstrating its ability to induce a pro-inflammatory phenotype in BV2 microglia [29].

In line with earlier experiments (Figure 2A), expression of *IL-1β* mRNA was significantly elevated in both VPAC1^+/−^ and VPAC2^+/−^ prior to exposure of cells to any inflammatory insults compared to WT control (#### *p* < 0.001; VPAC1^+/−^ and VPAC2^+/−^ vs. WT at 0 h post-LPS; Figure 7A), with significant difference between genotypes ($ *p* < 0.05 vs. VPAC1^+/−^ cells).

After 6 h exposure to LPS, the expression of *IL-1β* was significantly increased in WT (*** *p* < 0.001 vs. WT at 0 h; Figure 7A). There was no significant difference in the levels of *IL-1β* between WT and VPAC1^+/−^ at 6 h post-LPS, whereas there was a significant increase in LPS-treated VPAC2^+/−^ cells (## *p* < 0.01 vs. WT at 6 h post-LPS). At 12 h post-LPS, the expression of *IL-1β* was significantly increased in WT microglia (**** *p* < 0.0001 vs. WT at 0 h; Figure 7A). There was also a significant difference in the levels of *IL-1β* between WT and VPAC1^+/−^ (#### *p* < 0.0001 vs. VPAC1^+/−^ at 12 h post-LPS) and VPAC2^+/−^ cells (### *p* < 0.001 vs. VPAC2^+/−^ at 12 h post-LPS). *IL-1β* mRNA levels were significantly different between the two genotypes after 12 h of LPS ($$ *p* < 0.01). After 24 h of LPS, *IL-1β* expression was significantly increased in WT cells (**** *p* < 0.0001 vs. WT at 0 h; Figure 7A). In VPAC1^+/−^ cells, there was a statistically significant increase in *IL-1β* expression with respect to LPS-treated WT cells at the same time point (### *p* < 0.001 vs. WT after 24 h of LPS), as well as in VPAC2^+/−^ cells (# *p* < 0.05 vs. WT after 24 h of LPS). No significant differences were observed between mutant cells (*p* > 0.05).

*IL-6* mRNA was significantly upregulated in WT cells exposed to LPS after 6 (** *p* < 0.01 vs. WT at 0 h), 12, and 24 h (*** *p* < 0.001 at both time points, respectively; Figure 7B). Comparisons of *IL-6* mRNA levels across genotypes after 6 h of LPS treatment revealed no significant differences between WT and VPAC1^+/−^ cells. In contrast, *IL-6* expression was significantly elevated in VPAC2^+/−^ microglia compared to both WT cells (## *p* < 0.01) and VPAC1^+/−^ cells ($$ *p* < 0.01) under the same conditions. At 12 h post-LPS, there were no significant differences between WT and VPAC1^+/−^ cells, whereas a significant increase in *IL-6* expression was seen only in VPAC2^+/−^ cells (## *p* < 0.01 vs. WT after 12 h of LPS), which was also significant with respect to VPAC1^+/−^ cells ($$ *p* < 0.01 vs. VPAC1^+/−^ cells after 12 h of LPS). After 24 h of LPS stimulation, there was a significant difference in the levels of *IL-6* between WT and VPAC1^+/−^ cells (### *p* < 0.001 vs. WT at 24 h post-LPS), but no significant differences in VPAC2^+/−^ cells. A significant difference was found when comparing the *IL-6* gene expression between the heterozygous cell lines ($ *p* < 0.05 vs. VPAC1^+/−^ cells at 24 h post-LPS; Figure 7B).

A significant increase in *Iba1* mRNA expression was seen in both heterozygous cell lines with respect to WTs in the absence of LPS stimulation (### *p* < 0.01 vs. WT at 0 h; Figure 7C), with different mRNA expression between genotypes ($$ *p* < 0.01 vs. VPAC1^+/−^ at 0 h). After 6 h of LPS stimulation, the expression of *Iba1* was unchanged in WT cells but was increased in VPAC1^+/−^ cells (## *p* < 0.01 vs. WT at 6 h post-LPS) and more robustly in VPAC2^+/−^ cells (#### *p* < 0.0001 vs. WT at 6 h post-LPS). Direct comparisons between the two genotypes showed a significant increase in VPAC2^+/−^ vs. VPAC1^+/−^ cultures ($ *p* < 0.05 vs. VPAC1^+/−^ at 6 h post-LPS). At 12 h post-LPS, there was a significant upregulation of *Iba1* transcripts in WT microglia (* *p* < 0.05 vs. WT after 12 h of LPS). *Iba1* expression was significantly elevated in both VPAC1^+/−^ and VPAC2^+/−^ BV2 cells compared to WT after 12 h of LPS (#### *p* < 0.0001), with VPAC2^+/−^ cells showing higher levels than VPAC1^+/−^ ($$$ *p* < 0.001). By 24 h, *Iba1* expression in WT cells returned to baseline, while it remained significantly elevated in VPAC1^+/−^ and VPAC2^+/−^ cells (#### *p* < 0.0001 vs. WT), with VPAC2^+/−^ cells again showing a markedly higher expression than VPAC1^+/−^ ($$$$ *p* < 0.0001).

At baseline, *Arginase-1* mRNA expression was significantly reduced only in VPAC2^+/−^ cells (# *p* < 0.05 vs. WT cells at 0 h; Figure 7D), and there were no significant differences between haploinsufficient genotypes. After 6 h of LPS stimulation, the expression of *Arginase-1* was still not significant across genotypes. After 12 h, LPS induced a significant decrease in *Arginase-1* expression in WTs (* *p* < 0.05 vs. WT at 0 h). In comparison to WT cells, *Arginase-1* expression was only significantly elevated in VPAC2^+/−^ cultures (## *p* < 0.01 vs. WT at 12 h post-LPS). There were no differences in gene expression between the two mutant cell lines at the 12 h of LPS time point. At 24 h post-LPS stimulation, *Arginase-1* expression in WT was still significantly reduced with respect to the initial time point (* *p* < 0.05 vs. WT at 0 h). A significant increase in *Arginase-1* mRNAs was seen in VPAC2^+/−^ cells with respect to WT (## *p* < 0.01 vs. WT at 24 h post-LPS) and VPAC1^+/−^ cells at the same time point ($ *p* < 0.05 vs. VPAC1^+/−^ cells at 24 h post-LPS).

No significant differences in the expression of *CD206* were observed in VPAC1^+/−^ compared to WT microglia at baseline (*p* > 0.05 vs. WT at 0 h; Figure 7E). In VPAC2^+/−^ cells there was a significant increase in *CD206* mRNA expression (# *p* < 0.05 vs. WT at 0 h), which was also seen when comparing both genotypes ($ *p* < 0.05 vs. VPAC1^+/−^ cells). The expression of *CD206* in WT was significantly reduced by LPS exposure both at 6, 12, and 24 h (** *p* < 0.01 vs. WT at time 0 h for all the time points). At 6 h after LPS stimulation, there was a significant increase in the levels of *CD206* in VPAC1^+/−^ (## *p* < 0.01 vs. WT at 6 h post-LPS) and VPAC2^+/−^ cells (### *p* < 0.001). *CD206* expression was significantly increased in VPAC1^+/−^ and VPAC2^+/−^ cultures at 12 h post-LPS (# *p* < 0.05 vs. WT), with a more pronounced elevation at 24 h (## *p* < 0.01 vs. WT). No significant differences were observed between genotypes at 6, 12, or 24 h post-LPS.

### 3.7. VPAC2^+/−^ Microglia Release Increased NO in Response to a Lipopolysaccharide Challenge

**Figure 8:**
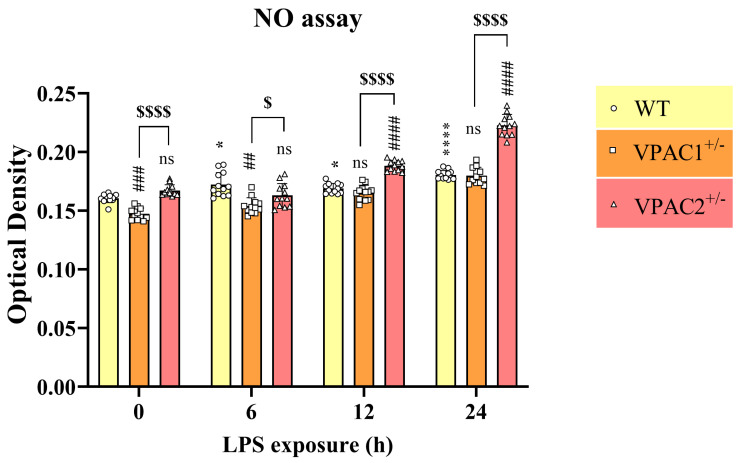
Nitric oxide (NO) release in lipopolysaccharide-stimulated WT, VPAC1^+/−^, and VPAC2^+/−^ BV2 microglia. Treatment causes increased nitric oxide (NO) release in VPAC2^+/−^ BV2 microglia. The bar graph depicts the time course of NO release in culture media after stimulation with 1 µg/mL LPS for 0, 6, 12, and 24 h, as determined using the Griess assay. Results shown are the mean ± SEM of three independent experiments, each using four separate biological replicates per genotype (n = 12). * *p* < 0.05 or **** *p* < 0.0001 vs. WT at time 0. ## *p*< 0.01, ### *p* < 0.001, or #### *p* < 0.0001 vs. WT at the corresponding time point. $ *p* < 0.05 or $$$$ *p* < 0.0001 vs. VPAC1^+/−^ cells. Statistical significance was determined using repeated measures two-way ANOVA, followed by Tukey post-hoc analysis. ns = not significant.

Lipopolysaccharide (LPS) is known to trigger a pro-inflammatory response associated with increased nitric oxide (NO) release and other inflammatory mediators in microglia [29,49]. While NO can be both protective and damaging, in this setting it served as a functional readout of LPS-induced inflammation. Based on our analyses suggesting that partial VPAC1 or VPAC2 gene ablation altered BV2 microglial responsiveness to inflammatory challenges, we sought to investigate if NO release was also affected. WT, VPAC1^+/−^, and VPAC2^+/−^ cells were either left untreated or exposed to LPS for varying times (0, 6, 12, and 24 h) to determine NO levels in culture media. As expected, in WT cultures, LPS treatment significantly increased NO release both at 6 h (* *p* < 0.05 vs. WT at time 0), 12 h (* *p* < 0.05), and more prominently at 24 h (**** *p* < 0.0001; Figure 8). In BV2 cultures with partial VPAC1 deletion, baseline NO levels were significantly less than in WT (### *p* < 0.001 vs. WT at the corresponding time); however, the difference was reduced after 6 h (## *p* < 0.01) to completely disappear at 12 and 24 h (ns, *p* > 0.05). When examining VPAC2^+/−^ cells, we did not identify significant changes of NO levels in either untreated or in LPS-treated cultures after 6 h (ns, *p* > 0.05), although levels were significantly higher than WT at later time points (#### *p* < 0.0001 vs. WT at both 12 and 24 h; Figure 8). Differences in NO release between VPAC1^+/−^ and VPAC2^+/−^ cells were consistent across all experimental time points ($$$$ *p* < 0.0001 vs. VPAC1^+/−^ cells at time 0, $ *p* < 0.05 at 6 h, and $$$$ *p* < 0.0001 at 12 and 24 h, respectively; Figure 8).

### 3.8. VPAC1 Heterozygosity Renders BV2 Cells More Susceptible to LPS-Induced Apoptosis

Since we established that VPAC haploinsufficiency triggered marginal changes in the baseline viability of BV2 microglia, our next approach was to investigate how these cells responded to an immunological challenge with LPS.

**Figure 9:**
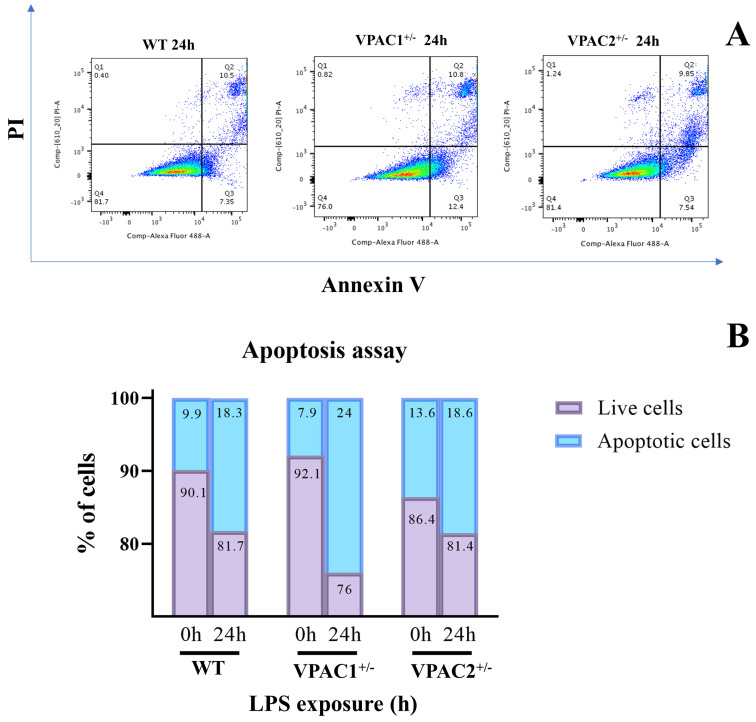
Effects of VPAC1 or VPAC2 haploinsufficiency on microglial cell viability following an LPS challenge. WT, VPAC1^+/−^, and VPAC2^+/−^ BV2 microglial cells were assessed for apoptosis using flow cytometry and Annexin V/propidium iodide (PI) staining after exposure to LPS for 24 h. (**A**) Scatter plots show the results obtained for each genotype after 24 h of LPS. (**B**) Quantification of the total % of live/dead cells. Exact percentages of apoptotic/live cells are indicated within each histogram for each genotype and represent the average of three independent determinations.

As anticipated, analysis of Annexin V/PI staining using flow cytometry revealed a statistically significant increase in the percentage of apoptotic cells after exposure of WT cells to LPS for 24 h (9.9% at 0 h vs. 18.3% at 24 h post-LPS [+8.4%]; Figure 9A,B). To our surprise, partial *VPAC1* gene loss rendered cells more susceptible to LPS-induced apoptosis, as the percentage of apoptotic cells increased from 7.9% (at baseline) to 24% after LPS exposure (+16.1%). In contrast, VPAC2^+/−^ cells demonstrated increased resilience to the detrimental effects of LPS, as the percentage of apoptosis increased from a baseline of 13.6% to 18.6% after 24 h of LPS (+5%) (Figure 9A,B).

### 3.9. VPAC1^+/−^ and VPAC2^+/−^ Microglia Display Opposite UPR Activation Patterns upon LPS Challenge

To explore the mechanisms underlying the opposite effects of VPAC1 and VPAC2 haploinsufficiency on cell survival at baseline and after LPS stimulation, we examined key UPR regulators in both genotypes under resting and LPS-treated conditions.

**Figure 10:**
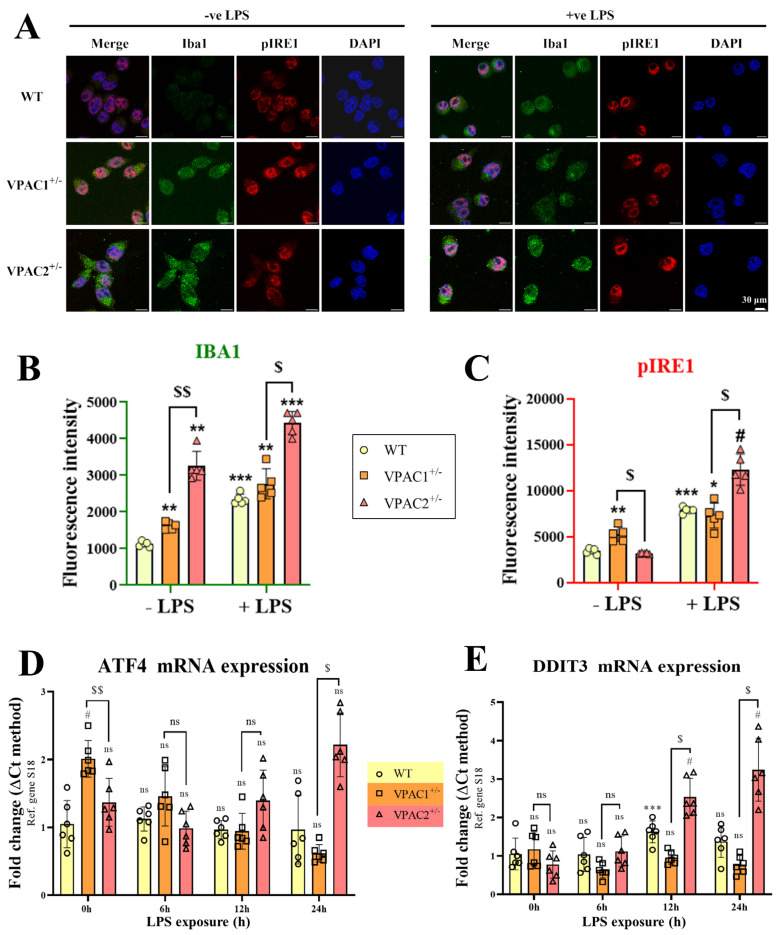
Opposing effects of VPAC1 and VPAC2 heterozygosity on the unfolded protein response (UPR) in unstimulated BV2 microglia. (**A**) Representative confocal images illustrating Iba1 and phospho-IRE1α^(Ser724)^ (pIRE1) co-staining in BV2 microglia from reagent control WT, VPAC1^+/−^, and VPAC2^+/−^ genotypes. (**B**) Quantification of Iba and (**C**) pIRE1 fluorescence intensity in untreated (-LPS) and LPS-treated cells (+LPS) from each genotype. The results shown are the mean ± SEM of at least n = 5 independent experiments. * *p* < 0.05, ** *p* < 0.01, and *** *p* < 0.001 vs. untreated WT cells. # *p* < 0.05 vs. LPS-treated cells or $ *p* < 0.05 and $$ *p* < 0.01 vs. VPAC1^+/−^ cells. Statistical analyses were performed using two-way ANOVA and Tukey post-hoc analyses. (**D**) Bar graphs depicting relative gene expression changes in *ATF4* and (**E**) *DDIT3* across genotypes ± SEM, as determined by real-time qPCR using n = 6 biological replicates per group. # *p* < 0.05 vs. WT and $ *p* < 0.05 vs. VPAC1^+/−^ cells, as determined by two-way repeated measures ANOVA and Tukey post-hoc test. ns = not significant, ATF4 = activating transcription factor 4, DDIT3 = DNA damage-inducible transcript 3, PERK = protein kinase R (PKR)-like endoplasmic reticulum kinase, and GAPDH = glyceraldehyde-3-phosphate dehydrogenase. ns = not significant.

As determined by co-immunohistochemistry (Iba1/pIRE1) and semi-quantification of immunoreactivity (Figure 10A–C), unstimulated WT microglia exhibited low Iba1 expression, whereas this was significantly increased in unstimulated VPAC1^+/−^ and VPAC2^+/−^ cells (## *p* < 0.01 vs. unstimulated WT for both; Figure 10B). However, Iba1 immunoreactivity was much more pronounced in VPAC2^+/−^ vs. VPAC1^+/−^ cultures ($$ *p* < 0.01 vs. unstimulated VPAC1^+/−^ cells). Post-LPS stimulation, Iba1 signals were remarkably increased in WT (*** *p* < 0.001 vs. unstimulated WT at time 0 h), VPAC1^+/−^ (* *p* < 0.05), and VPAC2^+/−^ cells (*** *p* < 0.001); however, the difference in signal intensity between the haploinsufficient cells was retained ($ *p* < 0.05 vs. unstimulated VPAC1^+/−^ cells).

Analyses of pIRE1 reactivity (an essential activator of the UPR) [46] highlighted a significant increase in pIRE1 staining intensity in unstimulated VPAC1^+/−^ cells with respect to WT controls (** *p* < 0.01 vs. unstimulated WT at time 0 h), whereas signal intensity was unaffected in VPAC2^+/−^ cells and significantly diminished with respect to VPAC1^+/−^ cells ($ *p* < 0.05 vs. VPAC1^+/−^ cells; Figure 10C). Upon LPS stimulation, the pattern was completely inverted. In fact, we observed a comparable level of pIRE1 immunoreactivity between WT and VPAC1^+/−^ cells, whereas this was robustly increased in VPAC2^+/−^ cells both with respect to LPS-stimulated WT (# *p* < 0.05 vs. post-LPS at 24 h) and VPAC1^+/−^ cells ($ *p* < 0.05; Figure 10C).

To corroborate these findings, we also determined the temporal regulation of *ATF4* and *DDIT3* in WT, VPAC1^+/−^, and VPAC2^+/−^ cells over an interval of 24 h (0, 6, 12, and 24 h). As shown, the heightened *ATF4* transcripts seen in unstimulated VPAC1^+/−^ cells (# *p* < 0.05 vs. unstimulated WT and $$ *p* < 0.01 vs. unstimulated VPAC2^+/−^ cells, respectively; Figure 10D) progressively returned to WT baseline levels within 6 h and remained stable under the 24 h timepoint (*p* > 0.05 vs. post-LPS WT cells at the corresponding timepoint; Figure 10D). In contrast, we reported a trend towards an increase in the expression of *ATF4* mRNAs in VPAC2^+/−^ cells starting from 12 h post-LPS stimulation, although the change became statistically significant only after 24 h ($ *p* < 0.05 vs. VPAC1^+/−^ cells).

Analysis of *DDIT3* transcripts showed a moderate increase in *DDIT3* expression in LPS-stimulated WT cells (*** *p* < 0.001 vs. unstimulated WT cells; Figure 10E). In VPAC1^+/−^ cells, LPS was demonstrated to be unable to induce *DDIT3* gene expression, as levels remained unchanged throughout the 24 h experimental window (Figure 10E). Conversely, we observed a significant induction of *DDIT3* gene expression in VPAC2^+/−^ cells both at 12 and 24 post-LPS exposure (# *p* < 0.05 vs. LPS-stimulated WT and $ *p* < 0.05 vs. LPS-stimulated VPAC1^+/−^ cells at both 12 and 24 h, respectively; Figure 10E).

### 3.10. Lipopolysaccharide Treatment Reduces BV2 Microglia Cell Motility Irrespective of Genotype

**Figure 11:**
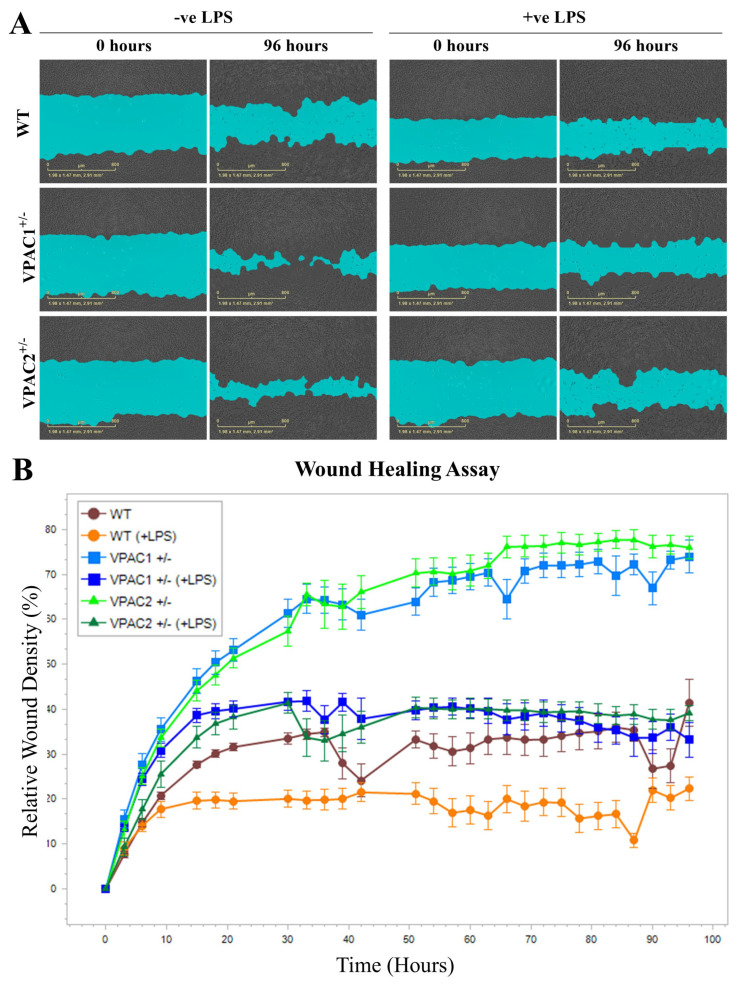
Effects of lipopolysaccharides on motility in WT, VPAC1^+/−^, and VPAC2^+/−^ BV2 microglia. (**A**) Representative images of the masked wound healing areas acquired using the Incucyte Cell Imager comparing initial and final wound size at time 0 and after 96 h for each genotype in unstimulated (−ve LPS) and stimulated cells (+ve LPS). (**B**) Line graph depicting the time course of relative wound density (RWD) ± SEM across unstimulated and stimulated cells with WT, VPAC1^+/−^, and VPAC2^+/−^ genotypes. Wounds were scanned at regular intervals of 3 h over 96 h. RWD measures the rate of wound closure compared to the initial scratch. As shown, LPS hindered cell motility, irrespective of genotypes. Mutant cells exhibited increased motility compared with WT cells, which was partly attenuated by LPS treatment. This experiment was performed on six independent batches of cells per genotype (n = 6).

Inflammatory conditions are known to impair microglial migration, particularly in vitro, where directional cues are absent [50]. We monitored real-time relative wound density (RWD) in WT, VPAC1^+/−^, and VPAC2^+/−^ BV2 microglia at baseline and following LPS exposure. Representative wound masks at 0 and 96 h, with and without LPS, are shown in Figure 11A.

Time-course analysis of RWD showed that VPAC1^+/−^ and VPAC2^+/−^ microglia were significantly more mobile than WT cells, with RWD values reaching 55–70% in mutants versus ~30% in WT cells (Figure 11B). Lipopolysaccharide (LPS) treatment induced a marked global reduction in RWD across the tested cell lines (mutant and WT), lowering it to approximately 40% in mutant cultures and below 20% in WT microglia (Figure 11B). Notably, despite the pronounced inhibitory effect of LPS on the motility of VPAC1^+/−^ and VPAC2^+/−^ microglia, the RWD values in haploinsufficient cultures remained relatively elevated and higher than in untreated WT cells.

## 4. Discussion

### 4.1. Defining Two Haploinsufficient Genotypes and the Essential Role of VPAC1 and VPAC2 Receptors in BV2 Microglial Homeostasis

Our study successfully established two distinct haploinsufficient BV2 microglial cell lines, VPAC1^+/−^ and VPAC2^+/−^, which revealed complementary yet functionally distinct roles of these receptors in maintaining microglial homeostasis under basal conditions and during LPS-induced pro-inflammatory-like phenotype. The partial loss of VPAC1 or VPAC2 was sufficient to induce significant biological changes, underscoring the importance of these receptors in modulating microglial function. Notably, despite repeated attempts, we were unable to generate homozygous knockout cell lines, suggesting that complete loss of either receptor may be incompatible with BV2 microglial cell viability. We also observed upregulation of VPAC1 and VPAC2 mRNAs in both VPAC1^+/−^ and VPAC2^+/−^ cells, which contrasts with expectations based on protein expression levels.

This inability to attain VPAC1^−/−^ or VPAC2^−/−^ mutants is unlikely to be merely a technical limitation but may instead reflect a critical dependence of BV2 microglia on VPAC receptor signaling for survival. Given the well-established roles of VPAC1 and VPAC2 in regulating cellular homeostasis [51], neuroprotection [13], immune responses [52], and survival [53], the absence of both alleles may disrupt essential intracellular pathways required for microglial maintenance. Regarding the unexpected upregulation of transcripts (but not protein levels) in haploinsufficient cell lines, this may reflect a compensatory transcriptional response triggered by loss of protein function: a phenomenon known as transcriptional adaptation or genetic compensation [54,55]. Additionally, CRISPR-Cas9 editing can unintentionally induce mRNA misregulation through mechanisms such as alternative splicing, activation of cryptic promoters, or partial escape from nonsense-mediated decay [35,56]. The concurrent increase in VPAC1 or VPAC2 mRNAs may similarly reflect a compensatory mechanism to preserve PACAP/VIP signaling, given their shared pathways and documented receptor cross-regulation [57,58]. Collectively, these findings highlight the indispensable nature of VPAC receptor signaling in microglial physiology, reinforcing their potential as key regulators of microglial function under both resting and inflammatory conditions.

### 4.2. VPAC Receptors and Their Role in the Unfolded Protein Response (UPR)

The unfolded protein response (UPR) is a highly conserved adaptive mechanism that maintains cellular homeostasis under conditions of endoplasmic reticulum (ER) stress [59]. It is primarily triggered by the accumulation of misfolded or unfolded proteins in the ER lumen, activating a series of signaling pathways aimed at restoring protein-folding capacity, reducing protein load, and, if homeostasis cannot be re-established, initiating programmed cell death [59,60,61]. The UPR is orchestrated by three primary ER stress sensors: protein kinase RNA-like ER kinase (PERK), inositol-requiring enzyme 1 (IRE1), and activating transcription factor 6 (ATF6), which act in concert to mitigate ER stress or drive apoptosis in cases of prolonged or excessive stress [45,62].

VPAC1 and VPAC2 receptors, which are class B G protein-coupled receptors (GPCRs) for vasoactive intestinal peptide (VIP) and pituitary adenylate cyclase-activating polypeptide (PACAP) [10,11], have been implicated in modulating cellular responses to stress [16,63], and in previous work we have established a potential crosstalk between these neuropeptide receptors and the ER stress machinery [42]. In this study, we harnessed an in vitro model of genetically engineered BV2 microglial cell lines to assess the role of VPAC receptors in the regulation of the UPR in cells under both baseline and stress conditions induced by LPS. Our findings provide novel insights into the receptor-specific roles of VPAC1 and VPAC2 in modulating UPR responses and highlight their differential contributions to cell viability and ER stress management.

### 4.3. Differential Baseline UPR Activation in VPAC1^+/−^ and VPAC2^+/−^ Cells

Our results demonstrate distinct baseline UPR activation patterns in VPAC1^+/−^ and VPAC2^+/−^ cells. Specifically, VPAC1^+/−^ cells exhibited heightened baseline expression of UPR markers, such as *ATF4* and pIRE1, compared to WT cells. This suggests that VPAC1 plays a crucial role in maintaining ER proteostasis under basal conditions. Increased pIRE1 activation in VPAC1^+/−^ cells further indicates an adaptive UPR response, promoting cellular resilience and reducing apoptotic susceptibility. These findings align with previous reports showing that VPAC1 signaling is integral to cellular stress responses [63,64].

Conversely, VPAC2^+/−^ cells displayed reduced levels of pIRE1 and PERK, with an accompanying increase in apoptotic cell death at baseline. This suggests that VPAC2 may suppress apoptosis under normal conditions by promoting the stabilization of ER proteostasis. However, the decreased activation of UPR sensors and the concurrent increase in the microglial marker Iba1 and that of other pro-inflammatory cytokines in untreated VPAC2^+/−^ cells raise the possibility that VPAC2’s role is more focused on immune regulation, potentially contributing to immune homeostasis under non-stressed conditions.

While the current study emphasizes the IRE1α and PERK branches, it is important to acknowledge that the UPR is a dynamic and multifaceted system comprising three major sensors: IRE1α, PERK, and *ATF6* [65]. Crosstalk between these pathways is crucial for determining cell fate decisions, particularly in microglia, where balancing pro-survival and pro-apoptotic signals influences neuroinflammation and repair [65,66]. For instance, the IRE1α pathway often supports adaptive responses, whereas PERK-ATF4 signaling may lead to either recovery or apoptosis, depending on the duration and severity of stress.

Our findings that baseline *DDIT3* gene expression is selectively reduced in VPAC2^+/−^ but not in VPAC1^+/−^ cells, suggest differential thresholds of UPR resolution. *DDIT3* encodes for a protein named CHOP, which is a well-established pro-apoptotic effector downstream of PERK [67]. Yet in certain contexts, it may also participate in re-establishing cellular homeostasis by regulating oxidative stress and autophagy [67,68]. Therefore, the observed differences in CHOP expression could reflect divergent adaptive strategies employed by microglia in response to VPAC1 vs. VPAC2 disruption. These distinctions underscore the need for a more nuanced interpretation of UPR activation beyond single-marker analyses.

### 4.4. Divergent LPS-Induced UPR Dysregulation in VPAC1^+/−^ and VPAC2^+/−^ Cells

The most striking observation in our study was the divergent UPR responses to LPS stimulation in VPAC1^+/−^ and VPAC2^+/−^ cells. LPS, a potent inducer of ER stress through Toll-like receptor 4 (TLR4) activation, triggered opposite UPR activation patterns in the two genotypes.

In VPAC1^+/−^ cells, LPS treatment led to a significant impairment in UPR activation, as indicated by reduced expression of *ATF4*, *DDIT3*, and pIRE1. This was accompanied by a marked increase in apoptotic cell death, suggesting that the absence of full VPAC1 signaling impairs the microglial cell’s ability to manage ER stress effectively. Our findings align with prior studies demonstrating that UPR dysregulation is associated with exacerbated apoptosis in microglia under stress conditions [69,70]. This highlights the importance of VPAC1 in promoting UPR signaling during inflammatory stress and underscores its role in maintaining cellular homeostasis under pathological conditions.

In contrast, VPAC2^+/−^ cells exhibited UPR activation following LPS exposure, characterized by increased expression of *ATF4*, *DDIT3*, and pIRE1. This enhanced UPR activation was associated with a global reduction in apoptotic cell death, suggesting that VPAC2 plays a protective role in maintaining cell viability during inflammatory stress. Interestingly, the elevated pIRE1 expression in VPAC2^+/−^ cells suggests that VPAC2 may interact with TLR4 signaling pathways to modulate UPR activation, a mechanism previously suggested in immune and neuronal cells [49,71]. This highlights the potential role of VPAC2 in orchestrating the UPR under inflammatory conditions, facilitating cell survival and stress resolution.

### 4.5. Cell Motility and Inflammatory Responses in VPAC1^+/−^ and VPAC2^+/−^ Cells

In addition to the UPR, we also assessed the migratory capacity of VPAC1^+/−^ and VPAC2^+/−^ BV2 microglia in response to LPS. Microglial motility is a key aspect of neuroinflammatory responses, and impaired migration is commonly observed under inflammatory conditions [50]. Our data revealed that both VPAC1^+/−^ and VPAC2^+/−^ cells exhibited enhanced motility at baseline compared to WT cells. However, LPS treatment significantly reduced the migratory capacity of both genotypes, with the greatest reduction observed in WT cells, which exhibited markedly lower motility compared to mutant cells. Despite the inhibitory effects of LPS on motility, the VPAC1^+/−^ and VPAC2^+/−^ cells retained higher migratory capacity, indicating a possible modulatory effect of VPAC receptor signaling on cell movement during inflammation.

These findings indicate that VPAC receptor signaling promotes microglial motility but is insufficient to counteract inflammation-induced migration deficits. The increased motility in mutant cells may reflect an elevated responsiveness to inflammatory cues, potentially advantageous in neuroinflammatory and neurodegenerative settings. However, it is important to note that proliferation assays were not performed in this study. As such, we cannot rule out the possibility that partial deletion of VPAC1 or VPAC2 may have affected cell growth, which could have contributed to the observed differences in wound closure independently or in addition to changes in motility. Future studies should incorporate proliferation analyses to disentangle the specific contributions of cell growth versus migration.

### 4.6. Study Limitations and Future Directions

One of the primary limitations of this study is the reliance on heterozygous KO models to infer the roles of VPAC1 and VPAC2 receptors in UPR regulation. While heterozygous deletion provides insights into receptor-specific effects, it does not fully account for potential compensatory mechanisms that may be activated in response to partial gene deletion. Additionally, the study does not assess whether the observed differences in UPR activation and apoptotic susceptibility are due to receptor-mediated signaling or secondary adaptations arising from long-term receptor deficiency and the possible effects of the interaction between microglia and other cellular subpopulations (i.e., astrocytes, neurons, etc.), as it happens in vivo. Future studies employing conditional or inducible knockout models would allow for a more precise dissection of VPAC receptor functions, minimizing compensatory effects and enabling temporal control of gene deletion. Moreover, pharmacological approaches using selective VPAC1 and VPAC2 agonists or antagonists could complement genetic models to validate receptor-specific contributions to ER stress regulation. Another limitation lies in the use of LPS as the sole stressor to investigate UPR dysregulation. While LPS is a well-established inducer of ER stress via TLR4 activation, its pro-inflammatory nature introduces confounding factors related to immune signaling that may obscure direct receptor-mediated effects. The absence of alternative stress paradigms, such as tunicamycin- or thapsigargin-induced ER stress, limits the generalizability of the findings to broader ER stress conditions. Future investigations should incorporate multiple ER stressors with distinct mechanisms of action to delineate the specificity of VPAC1 and VPAC2 modulation of the UPR. Additionally, exploring downstream transcriptional and translational changes using RNA sequencing or proteomics would provide deeper mechanistic insights into VPAC receptor-mediated regulation of ER stress and apoptosis.

Furthermore, while CRISPR-Cas9 was used to generate the edited VPAC1 and VPAC2 cell lines, whole-genome off-target analysis was not performed. However, all guide RNAs were designed using the Benchling platform, which calculates the likelihood of off-target cleavage. Only gRNAs with very low off-target probabilities were selected, providing high confidence in their specificity. Although this reduces the likelihood of unintended mutations, we acknowledge this as a technical limitation of the study.

A further consideration is the exclusive use of the BV2 murine microglial cell line in this study. Although BV2 cells are a widely accepted in vitro model due to their ease of use and reproducibility, they only partly capture the complex physiological and transcriptional landscape of primary microglia. This limitation may affect the translational relevance of our findings, particularly with respect to UPR activity, inflammatory responses, and apoptotic regulation.

To strengthen the translational impact, future studies should aim to validate the current findings using primary microglial cultures or more physiologically relevant systems, such as human inducible pluripotent stem cell (iPSC)-derived microglia or inducible/conditional knockout in vivo models. Incorporating such approaches will be crucial for establishing the broader applicability of VPAC receptor modulation in neuroinflammatory and neurodegenerative contexts.

## 5. Conclusions

Taken together, our findings suggest that VPAC1 and VPAC2 receptors exert distinct and context-dependent regulatory effects on UPR activation. Under homeostatic conditions, VPAC1 appears to be critical for maintaining ER proteostasis by promoting UPR activation and reducing apoptotic susceptibility. In contrast, VPAC2 appears to suppress apoptosis under basal conditions but plays a pivotal role in enhancing UPR activation during inflammatory stress.

These findings highlight the complexity of VPAC receptor signaling in ER stress regulation and suggest that their differential roles may have broader implications in neuroinflammatory and neurodegenerative diseases where ER stress and UPR dysregulation are key pathogenic factors. They also contribute valuable insights into the differential roles of VPAC1 and VPAC2 in microglial biology, providing a strong foundation for future investigations into the receptor-specific mechanisms governing microglial activation and neuroinflammatory processes. However, further mechanistic studies are warranted to elucidate the precise molecular interactions between VPAC signaling and UPR pathways.

## Figures and Tables

**Table 1:** Guide (g)-RNA sequences used for plasmid insertion into the appropriate genomic sequences of the indicated receptor genes. Predicted melting temperatures (Tm) are shown in the right column.

Gene Reference Sequence	Forward (5′-3′)	Tm (°C)	Location (Chromosome: Range)
	Reverse (3′-5′)		
Mouse VIPR1 (NC_000075.7)	(CACC)GTGCCAGCACGCAGAGCCAGC	83.9	9: 121471943–121471962
	(AAAC)GCTGGCTCTGCGTGCTGGCAC	80.5	
Mouse VIPR2 (NC_000078.7)	(CACC)GAGAGCTGCTAAGCAGCCAAA	74.5	12: 116043718–116043737
	(AAAC)TTTGGCTGCTTAGCAGCTCTC	68.5	

**Table 2:** PCR sequencing primers for gene knockout validation.

Gene Reference Sequence	Forward (5′-3′)	Location	Tm (°C)	Length (bp)
	Reverse (3′-5′)			
Mouse VIPR1 (NC_000075.7)	CCTGGAGCTGTGCCTCATAG	19	64.9	840
	TCTCAGAGGAGGTGACCAGG	839	64.6	
Mouse VIPR2 (NC_000078.7)	CACACCCACAGCCACTAAGA	96	63.9	991
	ACCATGACGGAGGCACAAAT	1067	66.6	

**Table 3:** Sanger sequencing primers used to verify the introduction of gene mutations.

Gene Reference (Accession Number)	Forward (5′-3′)	Location	Tm (°C)	Length (bp)
	Reverse (3′-5′)			
Mouse *VIPR1* (NC_000075.7)	CACAGACCCCGTAGGCG	45	65.6	677
	GGTCTAAGGTAGAGCAAGCCC	701	62.9	
Mouse *VIPR2* (NC_000078.7)	GAAAGGTGAAGCGTTGGATCT	267	64.3	601
	CAGAAACCTGAAGTCCCATTTTCC	844	67.5	

**Table 4:** Forward and reverse primers flanking the corresponding 5′ and 3′ regions of the respective coding sequences of the indicated target genes.

Gene	Forward (5′-3′)	Reverse (3′-5′)	Length (bp)
*VIPR1*	CCCTCTGTTTGGAGTTCACTAT	TACGACGAGTTCAAAGACCATT	88
*VIPR2*	ATTTCATAGATGCGTGTGGCTA	TGCTTCCTGTTGTAAGAGACAT	126
*IL-1β*	GCTACCTGTGTCTTTCCCGT	CATCTCGGAGCCTGTAGTGC	164
*IL-6*	CCCCAATTTCCAATGCTCTCC	CGCACTAGGTTTGCCGAGTA	141
*Iba1*	ACGTTCAGCTACTCTGACTTTC	GTTGGCCTCTTGTGTTCTTTG	107
*Arg1*	ACAAGACAGGGCTCCTTTCAG	TTAAAGCCACTGCCGTGTTC	105
*CD206*	AGTGATGGTTCTCCCGTTTC	ACCTTTCAGCTCACCACAAT	90
*ATF4*	CCTCAGACAGTGAACCCAAT	AATGCTCTGGAGTGGAAGAC	127
*DDIT3*	GCTCTCCAGATTCCAGTCAG	CTCCTTCTCCTTCATGCGTT	131
*TLR4*	ATCATGGCACTGTTCTTCTCC	ACTTTGCTGAGTTTCTGATCCA	107
*S18*	CCCTGAGAAGTTCCAGCACA	GGTGAGGTCGATGTCTGCTT	145

**Table 5:** Primary and secondary antibodies used for Western blot experiments.

Antibody	Source	Catalogue Number	Predicted Molecular Weight	Dilution
VPAC1	Merck Life Science	SAB4503084	51 kDa	1:1000
VPAC2	Abcam	ab28624	52 kDa	1:1000
Phospho-IRE1α (Ser724)	Merck Life Science	ZRB1072	110 kDa	1:2500
PERK	GeneTex	GTX129275	120 kDa	1:2000
GAPDH	Bio-Rad	VPA00187	37 kDa	1:1000
Goat Anti-Rabbit IgG H&L (HRP) (Secondary)	Abcam	ab6721	-	1:10,000

Abcam, Melbourne, VIC, Australia, GeneTex, Irvine, CA, USA.

**Table 6:** Primary and secondary antibodies used for immunocytochemistry.

Antibody	Source	Catalogue Number	Dilution
Phospho-IRE1α (Ser724)	Merck Life Science	ZRB1072	1:500
Ionized calcium-binding adapter molecule 1 (Iba1)	Merck Life Science	SAB2702364	1:250
Goat Anti-Rabbit IgG (H&L) (TRITC)	Abcam	ab6718	1:2500
Goat Anti-Mouse IgG (H&L) (FITC)	Abcam	ab6785	1:2500

## Data Availability

All data are reported in the published version of this article. Raw data can be made available upon reasonable request to the authors.

## Supplementary Materials

The following supporting information can be downloaded at https://www.mdpi.com/article/10.3390/cells14110769/s1; Figure S1: (A,B) Real-time monitoring of GFP+ cells demonstrate efficient transfection of both VPAC1- and VPAC2-containing plasmids after 24 h; Figure S2: (A,B) Validation of transient VPAC1 or VPAC2 gene knockdown in BV2 microglia; Figure S3: (A–G) Effects of transient VPAC1 or VPAC2 gene knockdown on the expression of pro-, anti-inflammatory and UPR genes in BV2 microglia.

## References

[1] DiSabato D.J., Quan N., Godbout J.P. (2016). Neuroinflammation: The devil is in the details. J. Neurochem..

[2] Tohidpour A., Morgun A.V., Boitsova E.B., Malinovskaya N.A., Martynova G.P., Khilazheva E.D., Kopylevich N.V., Gertsog G.E., Salmina A.B. (2017). Neuroinflammation and Infection: Molecular Mechanisms Associated with Dysfunction of Neurovascular Unit. Front. Cell. Infect. Microbiol..

[3] Jellinger K.A. (2010). Basic mechanisms of neurodegeneration: A critical update. J. Cell. Mol. Med..

[4] Sochocka M., Diniz B.S., Leszek J. (2017). Inflammatory Response in the CNS: Friend or Foe?. Mol. Neurobiol..

[5] Bjelobaba I., Savic D., Lavrnja I. (2017). Multiple Sclerosis and Neuroinflammation: The Overview of Current and Prospective Therapies. Curr. Pharm. Des..

[6] Heneka M.T., Carson M.J., El Khoury J., Landreth G.E., Brosseron F., Feinstein D.L., Jacobs A.H., Wyss-Coray T., Vitorica J., Ransohoff R.M. (2015). Neuroinflammation in Alzheimer’s disease. Lancet Neurol..

[7] Wang Q., Liu Y., Zhou J. (2015). Neuroinflammation in Parkinson’s disease and its potential as therapeutic target. Transl. Neurodegener..

[8] Kwon H.S., Koh S.-H. (2020). Neuroinflammation in neurodegenerative disorders: The roles of microglia and astrocytes. Transl. Neurodegener..

[9] Wofford K.L., Loane D.J., Cullen D.K. (2019). Acute drivers of neuroinflammation in traumatic brain injury. Neural. Regen. Res..

[10] Couvineau A., Ceraudo E., Tan Y.V., Nicole P., Laburthe M. (2012). The VPAC1 receptor: Structure and function of a class B GPCR prototype. Front. Endocrinol..

[11] Hirabayashi T., Nakamachi T., Shioda S. (2018). Discovery of PACAP and its receptors in the brain. J. Headache Pain.

[12] Dejda A., Sokolowska P., Nowak J.Z. (2005). Neuroprotective potential of three neuropeptides PACAP, VIP and PHI. Pharmacol. Rep..

[13] Soles-Tarres I., Cabezas-Llobet N., Vaudry D., Xifro X. (2020). Protective Effects of Pituitary Adenylate Cyclase-Activating Polypeptide and Vasoactive Intestinal Peptide Against Cognitive Decline in Neurodegenerative Diseases. Front. Cell. Neurosci..

[14] Dickson L., Finlayson K. (2009). VPAC and PAC receptors: From ligands to function. Pharmacol. Ther..

[15] Vaudry D., Falluel-Morel A., Bourgault S., Basille M., Burel D., Wurtz O., Fournier A., Chow B.K., Hashimoto H., Galas L. (2009). Pituitary adenylate cyclase-activating polypeptide and its receptors: 20 years after the discovery. Pharmacol. Rev..

[16] Nishimoto M., Miyakawa H., Wada K., Furuta A. (2011). Activation of the VIP/VPAC2 system induces reactive astrocytosis associated with increased expression of glutamate transporters. Brain Res..

[17] Musumeci G., Leggio G.M., Marzagalli R., Al-Badri G., Drago F., Castorina A. (2018). Identification of Dysregulated microRNA Networks in Schwann Cell-Like Cultures Exposed to Immune Challenge: Potential Crosstalk with the Protective VIP/PACAP Neuropeptide System. Int. J. Mol. Sci..

[18] Castorina A., Scuderi S., D’Amico A.G., Drago F., D’Agata V. (2014). PACAP and VIP increase the expression of myelin-related proteins in rat schwannoma cells: Involvement of PAC1/VPAC2 receptor-mediated activation of PI3K/Akt signaling pathways. Exp. Cell Res..

[19] Harmar A.J., Fahrenkrug J., Gozes I., Laburthe M., May V., Pisegna J.R., Vaudry D., Vaudry H., Waschek J.A., Said S.I. (2012). Pharmacology and functions of receptors for vasoactive intestinal peptide and pituitary adenylate cyclase-activating polypeptide: IUPHAR review 1. Br. J. Pharmacol..

[20] Maduna T., Lelievre V. (2016). Neuropeptides shaping the central nervous system development: Spatiotemporal actions of VIP and PACAP through complementary signaling pathways. J. Neurosci. Res..

[21] Couvineau A., Laburthe M. (2012). VPAC receptors: Structure, molecular pharmacology and interaction with accessory proteins. Br. J. Pharmacol..

[22] Constantinescu C.S., Farooqi N., O’Brien K., Gran B. (2011). Experimental autoimmune encephalomyelitis (EAE) as a model for multiple sclerosis (MS). Br. J. Pharmacol..

[23] Abad C., Jayaram B., Becquet L., Wang Y., O’Dorisio M.S., Waschek J.A., Tan Y.-V. (2016). VPAC1 receptor (Vipr1)-deficient mice exhibit ameliorated experimental autoimmune encephalomyelitis, with specific deficits in the effector stage. J. Neuroinflamm..

[24] Tan Y.V., Abad C., Wang Y., Lopez R., Waschek J. (2015). VPAC2 (vasoactive intestinal peptide receptor type 2) receptor deficient mice develop exacerbated experimental autoimmune encephalomyelitis with increased Th1/Th17 and reduced Th2/Treg responses. Brain Behav. Immun..

[25] Serpa A., Bento M., Caulino-Rocha A., Pawlak S., Cunha-Reis D. (2022). Opposing reduced VPAC(1) and enhanced VPAC(2) VIP receptors in the hippocampus of the Li(2+)-pilocarpine rat model of temporal lobe epilepsy. Neurochem. Int..

[26] Watson M.B., Nobuta H., Abad C., Lee S.K., Bala N., Zhu C., Richter F., Chesselet M.F., Waschek J.A. (2013). PACAP deficiency sensitizes nigrostriatal dopaminergic neurons to paraquat-induced damage and modulates central and peripheral inflammatory activation in mice. Neuroscience.

[27] Deczkowska A., Keren-Shaul H., Weiner A., Colonna M., Schwartz M., Amit I. (2018). Disease-Associated Microglia: A Universal Immune Sensor of Neurodegeneration. Cell.

[28] Goursaud S., Focant M.C., Berger J.V., Nizet Y., Maloteaux J.M., Hermans E. (2011). The VPAC2 agonist peptide histidine isoleucine (PHI) up-regulates glutamate transport in the corpus callosum of a rat model of amyotrophic lateral sclerosis (hSOD1G93A) by inhibiting caspase-3 mediated inactivation of GLT-1a. FASEB J..

[29] Karunia J., Niaz A., Mandwie M., Thomas Broome S., Keay K.A., Waschek J.A., Al-Badri G., Castorina A. (2021). PACAP and VIP Modulate LPS-Induced Microglial Activation and Trigger Distinct Phenotypic Changes in Murine BV2 Microglial Cells. Int. J. Mol. Sci..

[30] Thomas Broome S., Fisher T., Faiz A., Keay K.A., Musumeci G., Al-Badri G., Castorina A. (2021). Assessing the Anti-Inflammatory Activity of the Anxiolytic Drug Buspirone Using CRISPR-Cas9 Gene Editing in LPS-Stimulated BV-2 Microglial Cells. Cells.

[31] Castorina A., D’Amico A., Scuderi S., Leggio G., Drago F., D’Agata V. (2013). Dopamine D3 receptor deletion increases tissue plasminogen activator (tPA) activity in prefrontal cortex and hippocampus. Neuroscience.

[32] Schmittgen T.D., Livak K.J. (2008). Analyzing real-time PCR data by the comparative C(T) method. Nat. Protoc..

[33] Do J.H., Ko H.M., Suk K., Park E.J., Choi D.K. (2009). Genome-wide inspection of chromosomal aberrations in microglia BV-2 cells by array-based comparative genomic hybridization. BioChip J..

[34] Blasi E., Barluzzi R., Bocchini V., Mazzolla R., Bistoni F. (1990). Immortalization of murine microglial cells by a v-raf/v-myc carrying retrovirus. J. Neuroimmunol..

[35] Tuladhar R., Yeu Y., Tyler Piazza J., Tan Z., Rene Clemenceau J., Wu X., Barrett Q., Herbert J., Mathews D.H., Kim J. (2019). CRISPR-Cas9-based mutagenesis frequently provokes on-target mRNA misregulation. Nat. Commun..

[36] Delgado M., Jonakait G.M., Ganea D. (2002). Vasoactive intestinal peptide and pituitary adenylate cyclase-activating polypeptide inhibit chemokine production in activated microglia. Glia.

[37] Ohsawa K., Imai Y., Sasaki Y., Kohsaka S. (2004). Microglia/macrophage-specific protein Iba1 binds to fimbrin and enhances its actin-bundling activity. J. Neurochem..

[38] Lopez-Castejon G., Brough D. (2011). Understanding the mechanism of IL-1beta secretion. Cytokine Growth Factor Rev..

[39] Tanaka T., Narazaki M., Kishimoto T. (2014). IL-6 in inflammation, immunity, and disease. Cold Spring Harb. Perspect. Biol..

[40] Cherry J.D., Olschowka J.A., O’Banion M.K. (2014). Neuroinflammation and M2 microglia: The good, the bad, and the inflamed. J. Neuroinflamm..

[41] Tanaka S., Ohgidani M., Hata N., Inamine S., Sagata N., Shirouzu N., Mukae N., Suzuki S.O., Hamasaki H., Hatae R. (2021). CD206 Expression in Induced Microglia-Like Cells From Peripheral Blood as a Surrogate Biomarker for the Specific Immune Microenvironment of Neurosurgical Diseases Including Glioma. Front. Immunol..

[42] Withana M., Castorina A. (2023). Potential Crosstalk between the PACAP/VIP Neuropeptide System and Endoplasmic Reticulum Stress-Relevance to Multiple Sclerosis Pathophysiology. Cells.

[43] Mansouri S., Agartz I., Ögren S.O., Patrone C., Lundberg M. (2017). PACAP Protects Adult Neural Stem Cells from the Neurotoxic Effect of Ketamine Associated with Decreased Apoptosis, ER Stress and mTOR Pathway Activation. PLoS ONE.

[44] Miura A., Kambe Y., Inoue K., Tatsukawa H., Kurihara T., Griffin M., Kojima S., Miyata A. (2013). Pituitary adenylate cyclase-activating polypeptide type 1 receptor (PAC1) gene is suppressed by transglutaminase 2 activation. J. Biol. Chem..

[45] Walter P., Ron D. (2011). The unfolded protein response: From stress pathway to homeostatic regulation. Science.

[46] Li Y., Huang S., Wang J., Dai J., Cai J., Yan S., Huang Z., He S., Wang P., Liu J. (2022). Phosphorylation at Ser(724) of the ER stress sensor IRE1α governs its activation state and limits ER stress-induced hepatosteatosis. J. Biol. Chem..

[47] Guthrie L.N., Abiraman K., Plyler E.S., Sprenkle N.T., Gibson S.A., McFarland B.C., Rajbhandari R., Rowse A.L., Benveniste E.N., Meares G.P. (2016). Attenuation of PKR-like ER Kinase (PERK) Signaling Selectively Controls Endoplasmic Reticulum Stress-induced Inflammation Without Compromising Immunological Responses. J. Biol. Chem..

[48] Lively S., Schlichter L.C. (2013). The microglial activation state regulates migration and roles of matrix-dissolving enzymes for invasion. J. Neuroinflamm..

[49] Hines D.J., Choi H.B., Hines R.M., Phillips A.G., MacVicar B.A. (2013). Prevention of LPS-induced microglia activation, cytokine production and sickness behavior with TLR4 receptor interfering peptides. PLoS ONE.

[50] Zhang F., Nance E., Alnasser Y., Kannan R., Kannan S. (2016). Microglial migration and interactions with dendrimer nanoparticles are altered in the presence of neuroinflammation. J. Neuroinflamm..

[51] Castorina A., Scheller J., Keay K.A., Marzagalli R., Rose-John S., Campbell I.L. (2024). Increased Expression of the Neuropeptides PACAP/VIP in the Brain of Mice with CNS Targeted Production of IL-6 Is Mediated in Part by Trans-Signalling. Int. J. Mol. Sci..

[52] Nunan R., Sivasathiaseelan H., Khan D., Zaben M., Gray W. (2014). Microglial VPAC1R mediates a novel mechanism of neuroimmune-modulation of hippocampal precursor cells via IL-4 release. Glia.

[53] Castorina A., Tiralongo A., Giunta S., Carnazza M.L., Rasi G., D’Agata V. (2008). PACAP and VIP prevent apoptosis in schwannoma cells. Brain Res..

[54] Rossi A., Kontarakis Z., Gerri C., Nolte H., Hölper S., Krüger M., Stainier D.Y. (2015). Genetic compensation induced by deleterious mutations but not gene knockdowns. Nature.

[55] El-Brolosy M.A., Stainier D.Y.R. (2017). Genetic compensation: A phenomenon in search of mechanisms. PLoS Genet..

[56] Smits A.H., Ziebell F., Joberty G., Zinn N., Mueller W.F., Clauder-Münster S., Eberhard D., Fälth Savitski M., Grandi P., Jakob P. (2019). Biological plasticity rescues target activity in CRISPR knock outs. Nat. Methods.

[57] Langer I., Jeandriens J., Couvineau A., Sanmukh S., Latek D. (2022). Signal Transduction by VIP and PACAP Receptors. Biomedicines.

[58] Lu J., Piper S.J., Zhao P., Miller L.J., Wootten D., Sexton P.M. (2022). Targeting VIP and PACAP Receptor Signaling: New Insights into Designing Drugs for the PACAP Subfamily of Receptors. Int. J. Mol. Sci..

[59] Tsai Y.C., Weissman A.M. (2010). The Unfolded Protein Response, Degradation from Endoplasmic Reticulum and Cancer. Genes Cancer.

[60] Hasnain S.Z., Lourie R., Das I., Chen A.C., McGuckin M.A. (2012). The interplay between endoplasmic reticulum stress and inflammation. Immunol. Cell Biol..

[61] Almanza A., Carlesso A., Chintha C., Creedican S., Doultsinos D., Leuzzi B., Luis A., McCarthy N., Montibeller L., More S. (2019). Endoplasmic reticulum stress signalling—From basic mechanisms to clinical applications. FEBS J..

[62] Panganiban R.A., Park H.R., Sun M., Shumyatcher M., Himes B.E., Lu Q. (2019). Genome-wide CRISPR screen identifies suppressors of endoplasmic reticulum stress-induced apoptosis. Proc. Natl. Acad. Sci. USA.

[63] Langer I., Leroy K., Gaspard N., Brion J.-P., Robberecht P. (2008). Cell surface targeting of VPAC1 receptors: Evidence for implication of a quality control system and the proteasome. Biochim. Biophys. Acta (BBA) Mol. Cell Res..

[64] Storka A., Burian B., Führlinger G., Clive B., Sun T., Crevenna R., Gsur A., Mosgöller W., Wolzt M. (2013). VPAC1 receptor expression in peripheral blood mononuclear cells in a human endotoxemia model. J. Transl. Med..

[65] Hetz C. (2012). The unfolded protein response: Controlling cell fate decisions under ER stress and beyond. Nat. Rev. Mol. Cell Biol..

[66] Smith J.A., Turner M.J., DeLay M.L., Klenk E.I., Sowders D.P., Colbert R.A. (2008). Endoplasmic reticulum stress and the unfolded protein response are linked to synergistic IFN-beta induction via X-box binding protein 1. Eur. J. Immunol..

[67] Li Y., Guo Y., Tang J., Jiang J., Chen Z. (2014). New insights into the roles of CHOP-induced apoptosis in ER stress. Acta. Biochim. Biophys. Sin..

[68] Oyadomari S., Mori M. (2004). Roles of CHOP/GADD153 in endoplasmic reticulum stress. Cell Death Differ..

[69] James A.W., Bahader G.A., Albassan M., Shah Z.A. (2023). The ER chaperone, BIP protects Microglia from ER stress-mediated Apoptosis in Hyperglycemia. Neurochem. Int..

[70] Domínguez-Martín H., Gavilán E., Parrado C., Burguillos M.A., Daza P., Ruano D. (2024). Distinct UPR and Autophagic Functions Define Cell-Specific Responses to Proteotoxic Stress in Microglial and Neuronal Cell Lines. Cells.

[71] Kim J.A., Jang H.J., Hwang D.H. (2015). Toll-like receptor 4-induced endoplasmic reticulum stress contributes to impairment of vasodilator action of insulin. Am. J. Physiol. Endocrinol. Metab..

