# Supplementary material for: VPAC1 and VPAC2 Receptor Heterozygosity Confers Distinct Biological Properties to BV2 Microglial Cells
Source: Cells. 2025 May 23;14(11):769. doi: 10.3390/cells14110769 (PMC12153799; doi:10.3390/cells14110769)
Supplement: Supplementary file 1 [file cells-14-00769-s001.zip › cells-3569855-supplementary.pdf]

Article

VPAC1 and VPAC2 Receptor Heterozygosity Confers Distinct Biological Properties to BV2 Microglial Cells

Xin Ying Rachel Song [1], Margo Iris Jansen [1], Rubina Marzagalli [1], Giuseppe Musumeci [2], Velia D'Agata [2] and Alessandro Castorina [1,*]

[1] Laboratory of Cellular and Molecular Neuroscience, School of Life Sciences, Faculty of Science, University of Technology Sydney, Sydney, NSW 2007, Australia; raychelsxy@hotmail.com (X.Y.R.S.); margo.jansen@student.uts.edu.au (M.I.J.); rubina.marzagalli@uts.edu.au (R.M.)

[2] Department of Biomedical and Biotechnological Sciences, Section of Anatomy, Histology and Movement Sciences, University of Catania, 95100 Catania, Italy; g.musumeci@unict.it (G.M.); vdagata@unict.it (V.D.)

* Correspondence: alessandro.castorina@uts.edu.au

Supplementary Data

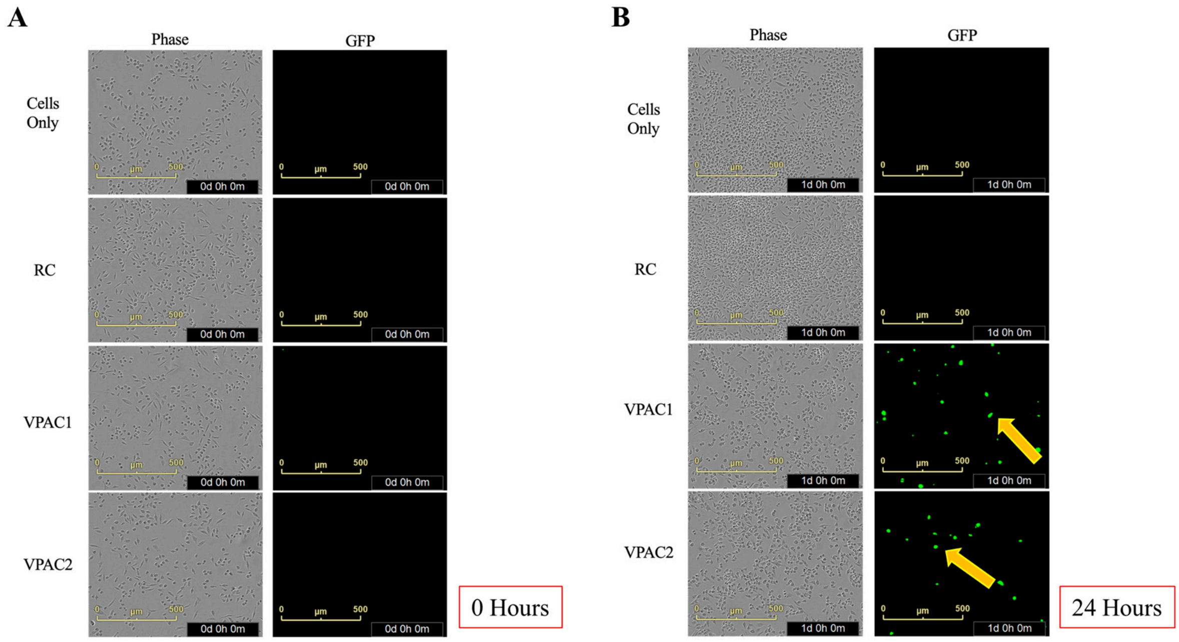

Figure S1. Real-time monitoring of GFP⁺ cells demonstrates efficient transfection of both VPAC1- and VPAC2-containing plasmids after 24 hours. Cells were seeded 5 × 10^4 cells per well in 12-well plates and transfected with the appropriate plasmids. (**A**, **B**) Representative bright light and fluorescent images of untreated cells (Cells Only), cells that were mock-transfected (reagent controls, RC) or cells transfected with the CRISPR-Cas9 plasmid to ablate the *VPAC1* or *VPAC2* receptor genes. GFP+ cells indicate effective integration of the plasmid (orange arrows).

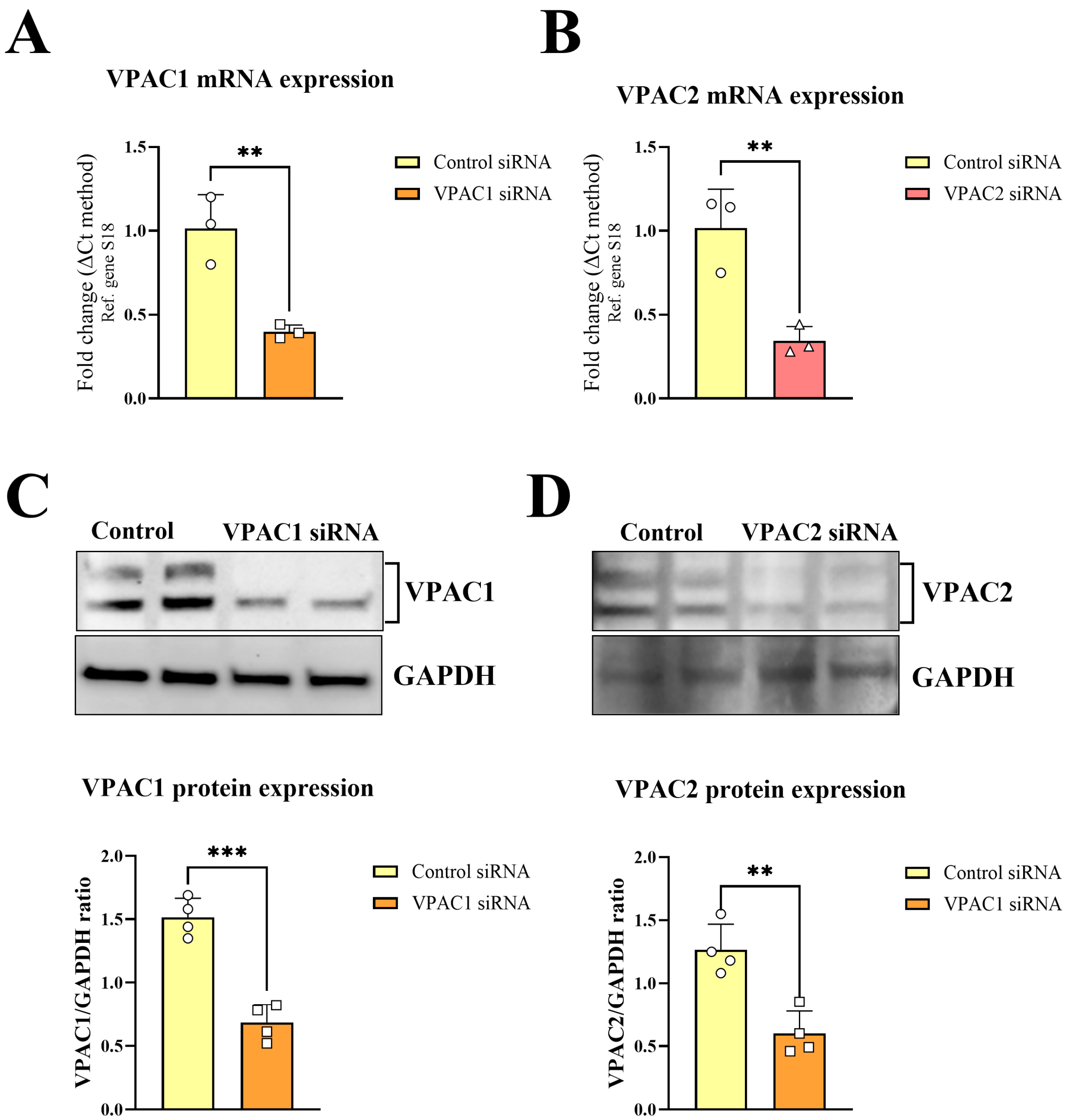

Figure S2. Transient silencing RNA (siRNA)-mediated attenuation of VPAC1 and VPAC2 gene and protein expression. BV2 cells were seeded in 6-well plates at a density of 2 × 10^5 cells per well in antibiotic-free DMEM supplemented with 10% fetal bovine serum and allowed to adhere overnight. Thereafter, cells were transfected as detailed in *Section 2.3 RNA Interference and Transfection of BV2 Microglial Cells*. Real-time qPCR analyses show a significant reduction of both (**A**) *VPAC1* and (**B**) *VPAC2* mRNAs in siRNA-transfected cells in comparison to untargeted siRNA controls (Control siRNA) (** $p < 0.01$ vs. Control siRNA). Data shown is the mean fold change ± SEM of two technical replicates from n = 3 biological replicates. Representative Western blot image of (**C**) VPAC1 and (**D**) VPAC2 protein expression in Control and VPAC1 or VPAC2 siRNA-treated BV2 cells. Relative bands' densities were normalized to the corresponding GAPDH, which was used as loading control. Graphs show the mean ± SEM of four biological replicates. ** $p < 0.01$ or *** $p < 0.001$ vs. Control siRNA, as determined by Student *T*-test.

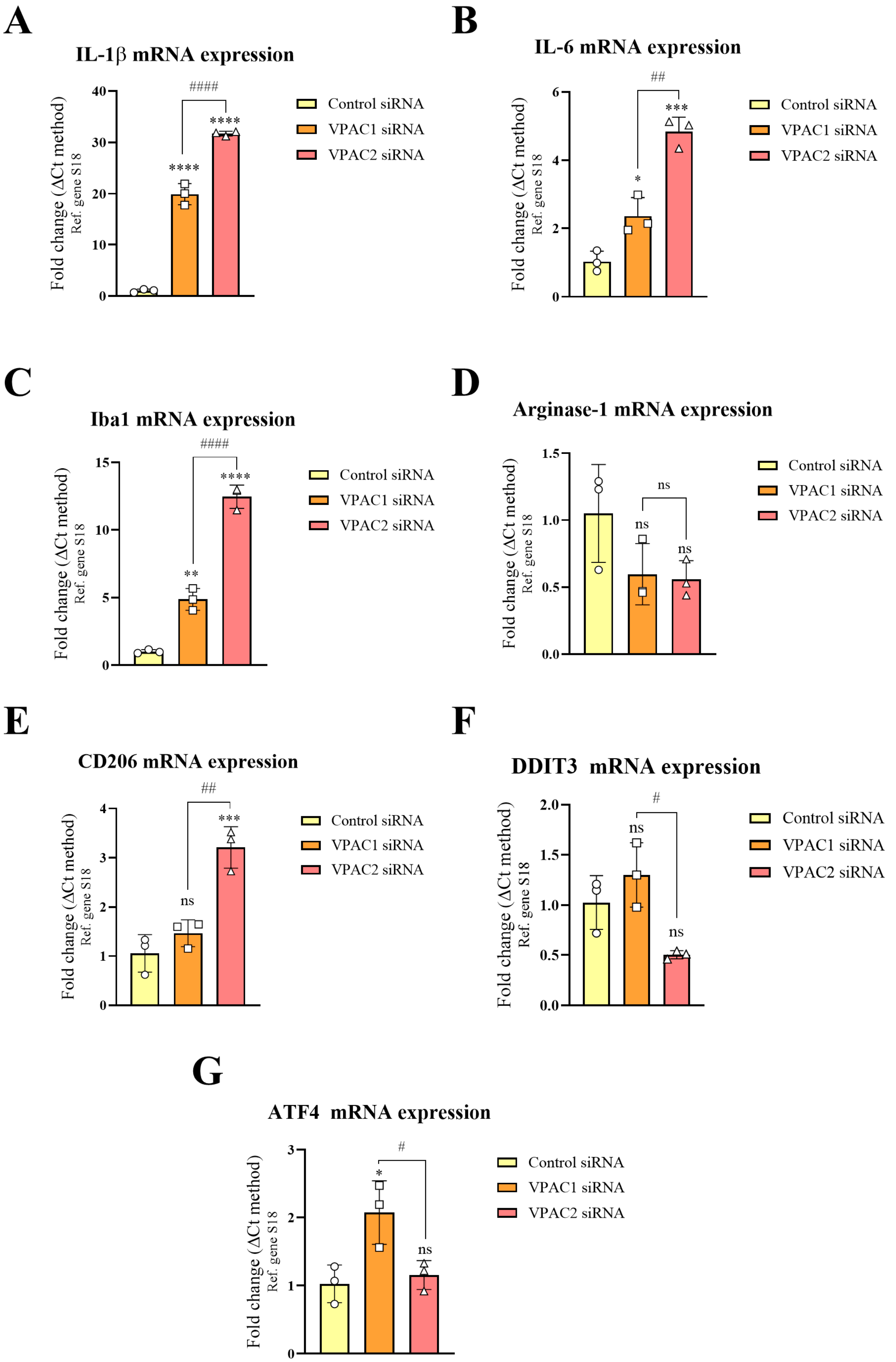

A
IL-1β mRNA expression
Fold change (ΔCt method)
Ref. gene S18
0
10
20
30
40
####
****
****
Control siRNA
VPAC1 siRNA
VPAC2 siRNA
B
IL-6 mRNA expression
Fold change (ΔCt method)
Ref. gene S18
0
2
4
6
##
*
***
Control siRNA
VPAC1 siRNA
VPAC2 siRNA
C
Iba1 mRNA expression
Fold change (ΔCt method)
Ref. gene S18
0
5
10
15
####
**
****
Control siRNA
VPAC1 siRNA
VPAC2 siRNA
D
Arginase-1 mRNA expression
Fold change (ΔCt method)
Ref. gene S18
0.0
0.5
1.0
1.5
ns
ns
ns
Control siRNA
VPAC1 siRNA
VPAC2 siRNA
E
CD206 mRNA expression
Fold change (ΔCt method)
Ref. gene S18
0
1
2
3
4
##
ns
***
Control siRNA
VPAC1 siRNA
VPAC2 siRNA
F
DDIT3 mRNA expression
Fold change (ΔCt method)
Ref. gene S18
0.0
0.5
1.0
1.5
2.0
#
ns
ns
Control siRNA
VPAC1 siRNA
VPAC2 siRNA
G
ATF4 mRNA expression
Fold change (ΔCt method)
Ref. gene S18
0
1
2
3
#
*
ns
Control siRNA
VPAC1 siRNA
VPAC2 siRNA

Figure S3. Gene expression of relevant pro-, anti-inflammatory and UPR genes in VPAC1- or VPAC2-siRNA transfected BV2 cells. Real-time qPCR analyses of (**A**) *IL-1β*, (**B**) *IL-6*, (**C**) *Iba1*, (**D**) *Arginase-1*, (**E**) *CD206*, (**F**) *DDIT3* and (**G**) *ATF4* in siRNA-transfected cells (*VPAC1* and *VPAC2* siRNAs) and untargeted siRNA controls (Control siRNA) As shown in panel **A**, transient *VPAC1* or *VPAC2* gene attenuation both increased *IL-1β* expression (**** $p < 0.0001$ *vs.* Control siRNA) which was more robust in *VPAC1* than *VPAC2* siRNA-treated cells (#### $p < 0.001$ *vs. VPAC1* siRNA). (**B**) *IL-6* mRNAs followed a similar pattern with a significant increase in cultures treated with VPAC1 siRNA (*$p<0.05$) and a more robust increase in cells treated with VPAC2 siRNA (*** $p < 0.001$), with an obvious difference between the two genotypes (## $p < 0.01$ *vs. VPAC1* siRNA). (**C**) *Iba1* mRNAs were also increased in cells with transient *VPAC1* gene attenuation (** $p < 0.01$) and more so in *VPAC2* siRNA cultures (**** $p < 0.0001$), with significant differences between the two (#### $p < 0.0001$). (**D**) Expression levels of the anti-inflammatory gene *Arginase-1* did not show any remarkable changes in response to transient gene knockdown of either *VPAC1* or *VPAC2* genes, whereas the expression of (**E**) another anti-inflammatory marker (*CD206*) was robustly induced in *VPAC2* knockdown cultures only (*** $p < 0.001$), which was also significantly increased with respect to *VPAC1* knockdown cells (## $p < 0.01$). (**F**) Expression of the UPR gene *DDIT3* showed no changes in *VPAC1* knockdown cells but significantly reduced expression in *VPAC2* knockdown cells (# $p < 0.05$). In contrast, (**G**) *ATF4* mRNAs were increased in *VPAC1* (* $p < 0.05$) but not in *VPAC2* knockdown cells, with significant differences between the two knockdown cultures (# $p < 0.05$). Results shown are the mean fold change ± SEM of two technical replicates from $n = 3$ biological replicates. * $p < 0.05$, ** $p < 0.01$, *** $p < 0.001$ and **** $p < 0.0001$ *vs.* Control siRNA. # $p < 0.05$, ## $p < 0.01$ and #### $p < 0.0001$ *vs. VPAC1* siRNA, as determined by one-way ANOVA followed by Tukey post-hoc test.
